# Locked nucleic acid (LNA) enhances binding affinity of triazole-linked DNA towards RNA†
†Electronic supplementary information (ESI) available. See DOI: 10.1039/c7cc05159j


**DOI:** 10.1039/c7cc05159j

**Published:** 2017-07-27

**Authors:** Pawan Kumar, Afaf H. El-Sagheer, Lynda Truong, Tom Brown

**Affiliations:** a Department of Chemistry , University of Oxford , 12 Mansfield Road , Oxford , OX1 3TA , UK . Email: tom.brown@chem.ox.ac.uk; b Chemistry Branch , Department of Science and Mathematics , Faculty of Petroleum and Mining Engineering , Suez University , Suez 43721 , Egypt

## Abstract

Oligonucleotides containing internal triazole–3′-LNA linkages bind to complementary RNA with similar affinity and specificity to unmodified oligonucleotides, and significantly better than oligonucleotides containing triazole alone. In contrast LNA on the 5′-side of the triazole does not stabilise duplexes. Triazole–LNA confers great resistance towards enzymatic degradation relative to LNA alone.

## Abstract 2

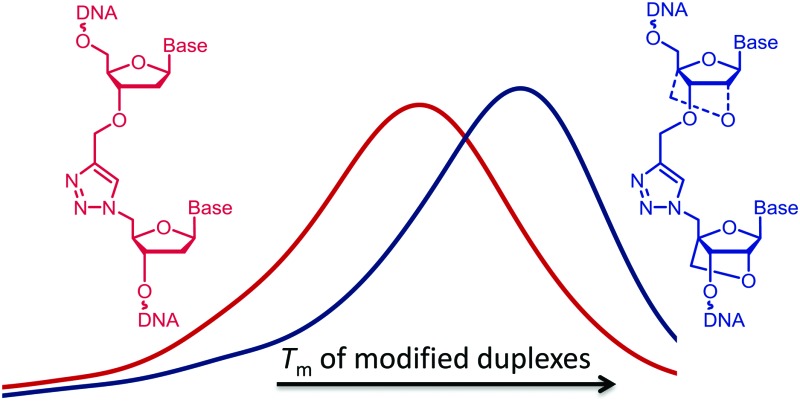
LNA improves the RNA-binding affinity and enzymatic stability of triazole-linked DNA.

## 

Oligonucleotides (ON's) containing triazole inter-nucleotide linkages have attracted considerable attention in the last decade.^1^–^6^ The most intensively studied of these is the biocompatible triazole-linkage in Fig. 1a which has recently emerged as an important tool in the chemical synthesis of long pieces of DNA.^7^ It is a mimic of natural phosphodiester-linked DNA and is functional in bacterial and human cells.^8^–^10^ However, ON's incorporating this linkage form less stable duplexes with complementary RNA/DNA targets compared to unmodified DNA strands.^11^,^12^ This makes them unfit for use as antisense ON's where high binding affinity for the target nucleic acid is essential. This problem was partially addressed by the introduction of an aminoethylphenoxazine nucleobase (G-clamp) on the 3′-side of the triazole linkage which significantly enhances the thermal stability of the modified duplex (Fig. 1b).^13^ However, G-clamp is mildly mutagenic^14^ and, being a mimic of 2′-deoxycytidine, it does not provide a solution for all nucleobase combinations. Recently, ON's featuring triazole-linked morpholino nucleotides (Fig. 1c) have been shown to hybridize to their RNA targets with slightly improved affinity compared to triazole alone.^15^ However, the resulting duplexes remain thermally less stable than their unmodified counterparts.

**Fig. 1:**
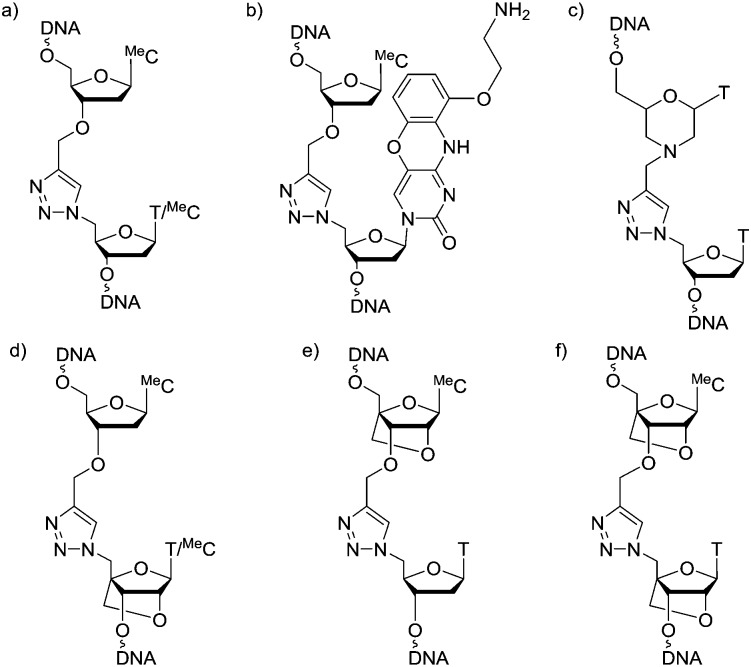
Modified DNA backbones. (a) Biocompatible triazole. (b) Triazole G-clamp. (c) Triazole-linked morpholino. (d) Triazole 3′-LNA. (e) Triazole 5′-LNA. (f) Triazole 3′,5′-LNA. T = thymin-1-yl, ^Me^C = 5-methylcytosin-1-yl.

Conformationally restricted locked nucleic acid (LNA) is one of the most promising classes of antisense ON's studied so far.^16^–^19^ ON's incorporating LNA bind to their complementary RNA targets with greatly improved affinity and selectivity; a single incorporation of LNA increases the melting temperature (*T*_m_) of a DNA:RNA duplex by up to 10 °C.^16^–^18^

Although ON's containing LNA units display some resistance to enzymatic degradation, they still possess a natural phosphodiester linkage which is vulnerable to nucleases. ON's incorporating both LNA and a triazole linkage, hereafter called triazole-linked LNAs, are particularly interesting as they should be highly resistant to degradation *in vivo*. In this study we aimed to improve the binding affinity of triazole linked ON's by introducing LNA sugars adjacent to the linkage. We show that ON's with a combination of LNA and triazole (Fig. 1d and f) bind to complementary RNA with affinity and selectivity comparable to unmodified ON's, and demonstrate that ON's of type 1f are highly resistant to nuclease digestion.

A number of triazole DNA backbones have been investigated previously.^20^ In this study we decided to focus on the biocompatible triazole in Fig. 1a because of its ease of synthesis.^14^

In initial studies we introduced LNA on one or both sides of the triazole linkage (Fig. 1d–f). Incorporation of LNA on 3′-side (Scheme 1A) is readily achieved using commercially available LNA phosphoramidites and conversion of the 5′-OH group to 5′-azide (Scheme 1A).^22^,^23^ Hence, LNA was incorporated as the last nucleotide at the 5′-end of the ON using phosphoramidite (**1**) where B is thymine. DMT-off ON's (**2**) were then treated with methyltriphenoxyphosphonium iodide in DMF whilst attached to the solid support,^22^ followed by reaction with sodium azide in DMF.^23^ Cleavage from the solid support and removal of all protecting groups gave ON's with a 5′-azido group (**3**). ON's carrying 3′-*O*-propargyl group were obtained using a commercially available solid support carrying 5′-*O*-(4,4′-dimethoxytrityl)-3′-*O*-propargyl-5-methyldeoxy-cytidine as the first nucleotide (**4**) as previously described.^8^,^24^ A Cu(i) catalysed alkyne azide cycloaddition (CuAAC) reaction^25^,^26^ of two short ON's, one with a 5′-azido group (**3**) and the other with a 3′-propargyl group (**4**), yielded ON's containing a single triazole linkage (Scheme 1A). This is a convenient synthetic strategy as it utilises simple commercially available intermediates.

**Scheme 1:**
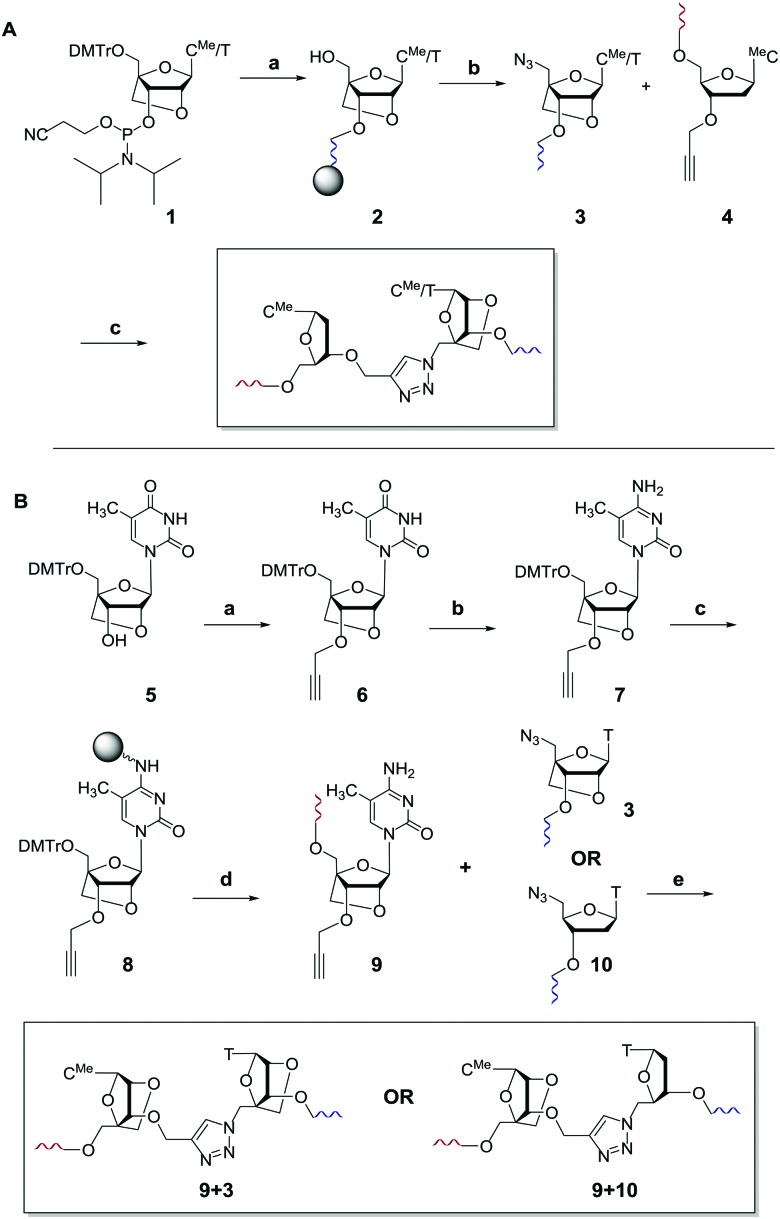
(A) Synthesis of triazole 3′-LNA. (a) Automated solid phase DNA synthesis. (b) 1. Methyltriphenoxyphosphonium iodide, DMF; 2. NaN_3_, DMF, 55 °C; 3. aqueous ammonia, 55 °C. (c) CuSO_4_, sodium ascorbate, tris(3-hydroxypropyltriazolylmethyl)amine.^21^ DMTr = 5′-*O*-(4,4′-dimethoxytrityl), ^Me^C = 5-methylcytosine. (B) Synthesis of triazole 5′-LNA and 5′,3′-LNA. (a) 1. NaH, DMF, 0 °C, 30 min, rt, 1 h; 2. propargyl bromide, 0 °C, 30 min, rt, 16 h, 79%. (b) 1. *N*-Methylimidazole, 0 °C, 15 min; 2. POCl_3_, 0 °C, 30 min, rt, 30 min. 3. Conc. NH_3_/H_2_O, rt, 16 h, 57%. (c) 1. Amino resin, succinic anhydride, DMAP, pyridine, rt, 20 h; 2. DIC, HOBT, pyridine, rt, 20 h; 3. pentachlorophenol, pyridine, rt, 3 h; 4. piperidine (10% in DMF), rt, 5 min; 5. capping with acetic anhydride, *N*-methylimidazole 1 : 1, rt, 1 h to give 26 μmol g^–1^ loading of nucleoside on resin. (d) Automated solid phase DNA synthesis. (e) CuSO_4_, sodium ascorbate, tris(3-hydroxypropyltriazolylmethyl)amine.^21^

The synthesis of ON's containing the 5′-LNA–triazole backbone was more complex (Scheme 1B), involving 3′-propargylation of 5′DMT–LNA thymidine (**5**) to give intermediate (**6**) which was converted to its cytidine analogue (**7**). This was attached to a solid support to give resin-bound 3′-propargyl LNA cytidine derivative (**8**) which was used in solid-phase ON synthesis. Click ligation reactions of ON (**9**) to ON's **3** and **10** yielded DNA constructs with 5′,3′-LNA–triazole and 5′-LNA–triazole backbones respectively. All ON's were purified by reversed-phase HPLC, and analysed by mass spectrometry (data in ESI†).

13-mer ON's containing a central ^Me^C–T step were synthesised. The ON sequence used was taken from our previous study.^13^ ON's were mixed with complementary DNA and RNA targets, and the thermal stabilities of the resulting duplexes were recorded by UV melting (Table 1). Interestingly, the thermal stability of the DNA:RNA duplex containing the triazole linkage with LNA on its 3′-side (ON2) was comparable to that of the unmodified duplex with ON1 (Δ*T*_m_ = –0.8 °C, Fig. 2). LNA significantly improved the stability of the modified DNA:RNA duplex relative to the duplex with only the triazole linkage (an increase of 5.4 °C in *T*_m_, compare ON2 with ON3, RNA target in Table 1). Thus, incorporation of LNA on the 3′-side of the triazole linkage counteracts the drop in the thermal stability caused by the triazole in the context of DNA:RNA duplexes. Duplexes containing a central ^Me^C–t–^Me^C step also showed similar trends (Table S2, ESI†). In contrast, 3′-LNA induced only a small increase of 2.9 °C in the thermal stability of dsDNA compared to the duplex containing only the triazole linkage (compare ON2 and ON3 with DNA target) and the stability of the triazole–LNA duplex was still very low compared to the unmodified dsDNA (ON1 *vs.* ON2, Δ*T*_m_ = –6.0 °C). For duplexes carrying no triazole linkage, LNA had the expected larger effect on binding to RNA targets (ON4, RNA target, Δ*T*_m_ = 6.1 °C) compared to DNA targets (ON4, DNA target Δ*T*_m_ = 3.3 °C). Preferential binding of LNA modified ON's for RNA targets is well known, and is due to the LNA sugar preferring the 3′-*endo* conformation.^16^,^17^ Surprisingly, the presence of LNA on the 5′-side of the triazole had no significant additional stabilising effect on DNA:RNA hybrids or DNA duplexes (Table 1, ON5 and ON6).

**Table 1:** Thermal melting (*T*_m_) data for duplexes containing a single triazole linkage

ON code	ON sequence (5′–3′)	DNA target		RNA target	
		*T*_m_ ^*a*^	Δ*T*_m_^*b*^	*T*_m_ ^*a*^	Δ*T*_m_^*b*^
ON1	CGACG^Me^CTTGCAGC	64.2		62.8	
ON2	CGACG^Me^C**t**T^**L**^TGCAGC	58.2	–6.0	62.0	–0.8
ON3	CGACG^Me^C**t**TTG CAGC	55.3	–8.9	56.6	–6.2
ON4	CGACG^Me^CT^**L**^TGCAGC	67.5	+3.3	68.9	+6.1
ON5	CGACG^Me^C^**L**^**t**TTGCAGC	52.7	–11.5	55.5	–7.2
ON6	CGACG^Me^C^**L**^**t**T^**L**^TGCAGC	58.4	–5.8	62.9	+0.1

^*a*^Melting temperatures (*T*_m_) were obtained from the maxima of the first derivatives of the melting curves (*A*_260_*vs.* temperature) recorded in a buffer containing 10 mM phosphate and 200 mM NaCl at pH 7.0 using 3.0 μM concentrations of each strand.

^*b*^Δ*T*_m_ = change in *T*_m_ for a modified duplex relative to the unmodified duplex. T^**L**^ is LNA thymidine, ^Me^C is 5-methylcytosine and **t** is a triazole linkage (Fig. 1a). DNA target: 5′-dGCT GCA AGC GTC G. RNA target: 5′-rGCU GCA AGC GUC G.

**Fig. 2:**
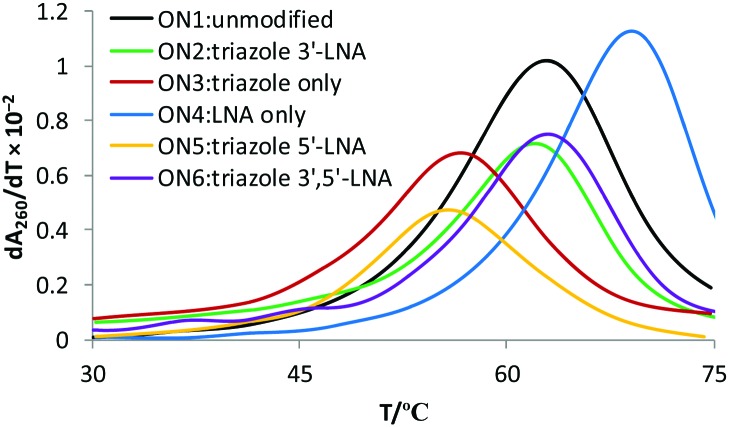
UV melting studies (derivatives of melting curves). DNA:RNA hybrid duplex containing a triazole linkage are stabilized by the introduction of LNA on the 3′-side or on both (3′- and 5′-) sides of the triazole linkage (compare ON2 with ON3 and ON6). For sequences see Table 1.

For therapeutic oligonucleotides improved thermal stability must also be accompanied by efficient mismatch discrimination. The ability of the studied ON's to discriminate between matched and mismatched RNA strands was assessed by mixing them with targets containing a mismatch nucleotide opposite the thymine nucleobase on 3′-side of the triazole linkage (T–X mismatch where X = C, T or G). The ON's containing triazole-linked 3′-LNA were found to maintain the fidelity of Watson–Crick base pairing, and effectively discriminated against mismatched targets with efficiency parallel to that of unmodified ON's (Table S3, ESI†).

Next, ON's incorporating two triazole inter-nucleotide linkage steps were prepared by templated CuAAC click ligation reactions in the presence of a complementary splint (procedure in ESI†). The ligated ON's were purified by denaturating 20% polyacrylamide gel electrophoresis and were evaluated for their binding affinity for complementary DNA/RNA strands (Table 2). Pleasingly, ON's containing two triazole–3′-LNA-linkages (^Me^C–T steps) showed a significant improvement in binding affinity for their RNA targets relative to ON's incorporating two triazole linkages without 3′-LNA (an increase of 5.0 °C/modification in *T*_m_, compare ON8 and ON9, RNA target). When compared to unmodified ON7, a drop of only 0.8 °C/modification was observed (ON8, RNA target). These stability studies suggest that DNA:RNA duplexes can tolerate multiple LNA–triazole linkages, which is not feasible for triazole linkages alone due to the greater lowering of *T*_m_. Since the improvement in binding affinity is specific for DNA:RNA hybrids, triazole-linked LNA could find use in selective probes for RNA targeting. ON's incorporating two ^Me^C–t–^Me^C steps showed similar trends (Table S4, ESI†).

**Table 2:** Thermal melting (*T*_m_) data for duplexes incorporating two triazole linkages

ON code	ON sequence (5′–3′)	DNA target		RNA target	
		*T*_m_ ^*a*^	Δ*T*_m_/mod^*b*^	*T*_m_ ^*b*^	Δ*T*_m_/mod^*b*^
ON7	CGA^Me^CTTCT^Me^CTAGC	57.1		58.8	
ON8	CGA^Me^C**t**T^**L**^TCT^Me^C**t**T^**L**^AGC	48.0	–4.5	57.1	–0.8
ON9	CGA^Me^C**t**TTCT^Me^C**t**TAGC	42.3	–7.4	47.1	–5.8
ON10	CGA^Me^CT^**L**^TCT^Me^CT^**L**^AGC	62.2	+2.5	70.0	+5.6

^*a*^Melting temperatures (*T*_m_) were obtained from the maxima of the first derivatives of the melting curves (*A*_260_*vs.* temperature) recorded in a buffer containing 10 mM phosphate and 200 mM NaCl at pH 7.0 using 3.0 μM concentrations of each strand.

^*b*^Δ*T*_m_ = change in *T*_m_ for a modified duplex relative to the unmodified duplex. T^**L**^ is LNA thymidine, ^Me^C is 5-methylcytosine and **t** is a triazole linkage (Fig. 1a). DNA target; 5′-dGCT AGA GAA GTC G. RNA target; 5′-rGCU AGA GAA GUC G.

The global structures of the modified duplexes were also studied by CD-spectroscopy (ESI,† Fig. S5 and S6). Both modified and unmodified duplexes showed similar CD spectra suggesting that neither LNA nor triazole-linkage induced any significant change in the global geometry of the studied duplexes.

3'-Exonuclease stability studies using snake venom phosphodiesterase 1 (SVPD, from *Crotalus adamanteus* venom) showed that the combination of triazole and 3′-LNA is more resistant to degradation than unmodified ON's or those containing only LNA (Fig. S7, ESI†), and the combination of 5′-LNA–triazole–3′LNA was highly stabilising (Fig. 3). Evidence for the enzyme pausing at the modified backbone linkage is clearly visible (Fig. 3 lane 12). The presence of the triazole seems to protect the unmodified nucleotides on its 3′-side possibly by reducing binding to the enzyme. This encouraging result warrants more detailed investigation. It suggests that ON's containing multiple triazole–LNA linkages will have significant *in vivo* stability.

**Fig. 3:**
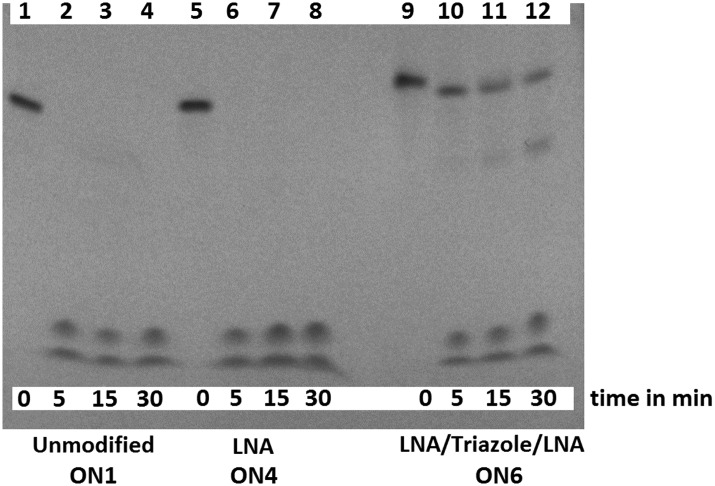
LNA triazole stabilises ON's to 3′-exonuclease digestion. The unmodified ON (lanes 2–4) and LNA ON (lanes 6–8) were fully digested within 5 min whereas the LNA–triazole–LNA ON was still visible after 30 min (lane 12).

Finally, we set out to see if the triazole-linkage in combination with LNA at its 3′-side can be read through by DNA polymerases. To evaluate this, an 81-mer PCR template containing triazole LNA was prepared by a splint assisted CuAAC click ligation reaction (ESI†). PCR amplification of this modified template was achieved using Gotaq DNA polymerase (Fig. 4). The PCR reaction requires a long extension time for first few cycles (5 min), in agreement with a previous report of LNA-modified templates being amplified by PCR.^27^ The amplicon was shown by agarose gel electrophoresis and mass spectrometry to be the fully extended product. A linear copying experiment for the same template using DNA polymerase 1, large Klenow fragment and a reaction time of 2.5 h also gave a fully extended product. Although this extension time is longer than required for templates with only a triazole linkage^8^ (no LNA) it demonstrates that the combination of LNA and triazole can be reliably read through by DNA polymerases.

**Fig. 4:**
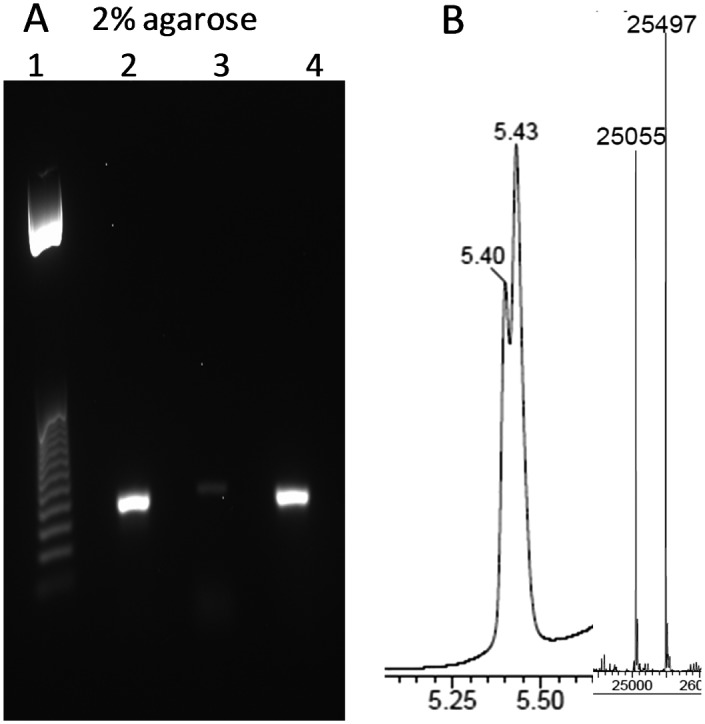
LNA triazole DNA template is correctly amplified by PCR. (A) 2% agarose gel using template GCA TTC GAG CAA CGT AAG ATC G^Me^CtT^**L**^ AGC ACA CAA TCT CAC ACT CTG GAA TTC ACA CTG ACA ATA CTG CCG ACA CAC ATA ACC where t represents triazole linkage and T^L^ is LNA thymidine. Lane 1; 25 bp ladder. Lane 2; PCR reaction using modified template. Lane 3; negative control, PCR reaction with primers but no template. Lane 4; positive control, PCR reaction with unmodified template (for sequence see ESI†). (B) UV trace from HPLC of HPLC/mass spec and ESI mass spectrum of the PCR product (both strands). [M + A] strand 1: calc. 25053, found 25055. Strand 2: calc. 25496, found 25497.

In summary, the synthesis and properties of ON's incorporating internal triazole–LNA linkages are reported. Introduction of LNA directly at the 3′-side of the triazole significantly improves the thermal stability of DNA:RNA duplexes, and ON's carrying one or two such triazole–LNA linkages display RNA binding affinity close to that of unmodified ON's. In contrast, duplex stabilisation is not observed when LNA is positioned at the 5′-side of the triazole. This leads us to the following hypothesis: it is known that LNA stabilises DNA:RNA duplexes by reducing phosphate backbone flexibility and inducing the A-conformation (3′-endo sugar). This property is preserved in ON's containing triazole–3′-LNA because the LNA sugar is attached to the natural phosphodiester group. In contrast, in triazole–5′-LNA the locked sugar is attached to a non-optimal triazole linkage and the influence of LNA is lost. This suggests that LNA will generally stabilise DNA:RNA hybrids containing other triazole backbone analogues in the DNA strand, provided that the locked sugar is on the 3′-side of the modified backbone. In contrast, LNA on the 5′-side is unlikely to be stabilising unless the modified backbone is a very close isostere of a natural phosphodiester.

In a related paper in this issue by Watts *et al.*^28^ the triazole-linked ribo and xylo-LNA gave high target RNA binding affinity when incorporated at the 3′ or 5′ termini of siRNAs, but extremely low binding affinity at internal positions. The triazole moiety was attached to the LNA sugars by more rigid linkers than the one studied here, and these results are not inconsistent with the above hypothesis.

Importantly, combining triazole and LNA in a single compact unit, as achieved here, maximises the number of triazole–LNA linkages that can be incorporated into oligonucleotides. We have shown that the combination of LNA and triazole is highly resistant to 3′-exonuclease digestion; hence multiple additions are expected to greatly increase *in vivo* stability of therapeutic ON's. They might also have implications for delivery of modified ON's into cells due to their reduced overall negative charge. To this end we are currently synthesising dinucleotide triazole–LNA phosphoramidite monomers to facilitate the assembly of such densely modified ON's.

This work was supported by UK BBSRC grants BB/J001694/2 (extending the boundaries of nucleic acid chemistry), a Marie Sklodowska-Curie Individual Fellowship (project; INAME) to PK from the European Commission and the intramural program of the National Heart, Lung and Blood Institute, NIH, USA through the NIH-Oxford-Cambridge Research Scholars Program (LT).

## Supplementary Material

Supplementary information
