# Supplementary material for: Locked nucleic acid (LNA) enhances binding affinity of triazole-linked DNA towards RNA
Source: Chem Commun (Camb). 2017 Jul 27;53(63):8910–3. doi: 10.1039/c7cc05159j (PMC5708354; doi:10.1039/c7cc05159j)
Supplement: Supplementary file 1 [file CC-053-C7CC05159J-s001.pdf]

Electronic Supplementary Material (ESI) for Chemical Communications.
This journal is © The Royal Society of Chemistry 2017

Supporting Information

Locked Nucleic Acid (LNA) enhances binding affinity of triazole-linked DNA towards RNA

Pawan Kumar,[a*] Afaf H. El-Sagheer[a,b] Lynda Truong[a] and Tom Brown[a*]

[a]Department of Chemistry, University of Oxford, 12 Mansfield Road, Oxford, OX1 3TA, UK, [b]Chemistry Branch, Department of Science and Mathematics, Faculty of Petroleum and Mining Engineering, Suez University, Suez 43721, Egypt

Email: tom.brown@chem.ox.ac.uk

Table of Contents

General synthetic Procedures 2

Synthesis of 5′-*O*-(4,4′-dimethoxytrityl)-3′-*O*-propargyl-LNA thymidine (6) 2

Synthesis of 5′-*O*-(4,4′-dimethoxytrityl)-3′-O-propargyl-5-methyl LNA cytidine (7) 3

Preparation of solid support carrying 5′-*O*-(4,4′-dimethoxytrityl)-3′-O-propargyl-5-methyl LNA cytidine (8) 3

Synthesis and Purification of oligonucleotides 4

Synthesis of DNA oligonucleotides 4

Synthesis of RNA oligonucleotides 4

Removal of 2′-TBS protection of RNA oligonucleotides 5

Purification of oligonucleotides (DNA or RNA) 5

Synthesis of 3′-alkyne-5-methyl dC and 3′-alkyne-5-methyl LNA cytidine oligonucleotides 5

Synthesis of 5′-azide modified oligonucleotides 5

Synthesis of 13-mer oligonucleotides incorporating a single triazole linkage 6

Synthesis of 13-mer oligonucleotides incorporating two triazole linkages 6

Synthesis of an 81-mer template incorporating a single LNA-triazole linkage 6

Table S1. Mass spec analysis of modified oligonucleotides 6

Ultraviolet melting studies 7

Additional T_m data 8

CD spectroscopy 9

Snake venom phosphodiesterase stability 10

Linear copying of an 81-mer template incorporating a single LNA-triazole linkage 11

PCR of an 81-mer template incorporating a single LNA-triazole linkage 11

UV trace from HPLC of HPLC/mass spec for modified oligonucleotides 12

NMR Spectra 18

General synthetic Procedures:

All reagents were purchased from Sigma-Aldrich, Alfa Aesar, Fisher Scientific, or Link Technologies and used without further purification. Pyridine (from KOH) and $POCl_3$ were freshly distilled before use, and THF was obtained using the MBraun SPS Bench Top solvent purification system (SPS). All air/moisture sensitive reactions were carried out under inert atmosphere (argon) in oven-dried glassware. Reactions were monitored by thin layer chromatography (TLC) using Merck Kieselgel 60 F24 silica gel plates (0.22 mm thickness, aluminium backed). The compounds were visualized by UV irradiation at 254/265 nm and by staining in *p*-anisaldehyde solution. Column chromatography was carried out under pressure (Flash Master Personal) using Biotage Isolute SPE columns. Columns were primed with CH_2Cl_2 containing 1% pyridine prior to use. ^{1}H and ^{13}C spectra were measured on a Bruker AVII 500 spectrometer at 500 MHz and 126 MHz, respectively. Chemical shifts are given in ppm and were internally referenced to the appropriate residual solvent signal, all coupling constants (*J*) are quoted in Hertz (Hz). Assignment of compounds was aided by COSY, HSQC, HMBC, and DEPT-135 experiments. High-resolution mass spectra were measured on a Bruker 9.4 FT-ICR-MS mass spectrometer, and samples were run in MeOH.

Synthesis of 5′-*O*-(4,4′-dimethoxytrityl)-3′-*O*-propargyl-LNA thymidine (6)

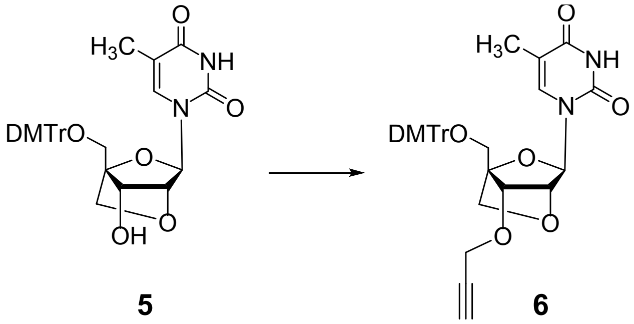

Nucleoside **5**[1] (2.00 g, 3.50 mmol) was co-evaporated with anhydrous THF (3 × 15 mL) and re-dissolved in anhydrous THF (24 mL). The solution was cooled to 0 °C and NaH (60% suspension in mineral oil, 0.348 g, 14.5 mmol) was added in portions over 5 min. The reaction mixture was stirred on ice for 30 min and at room temperature for 1 h. Propargyl bromide (0.374 mL, 4.20 mmol) was added at 0 °C and the reaction was stirred on ice for 30 min and at room temperature for 16 h. Solvent was removed at reduced pressure and the residue was dissolved in EtOAc (100 mL) and washed with brine (2 × 50 mL). The organic phase was dried (Na_2SO_4) and concentrated and the resulting crude was purified using column chromatography (EtOAc in hexane, 10% to 80%, v/v) to obtain compound **6** (1.68 g, 2.75 mmol, 79%) as a white foam. R_f = 0.4 (70% EtOAc in hexane, v/v). ESI HRMS m/z 633.2208 ([M+Na]$^+$, $C_{35}H_{34}O_8N_2Na^+$ calc. 633.2207. ^{1}H NMR (500 MHz, DMSO-*d*$_6$) δ 11.47 (s, 1H, NH), 7.59 (d, *J* = 1.1 Hz, 1H, H-6), 7.46 – 7.45 (m, 2H, DMTr), 7.36 – 7.31 (m, 6H, DMTr), 7.28 – 7.25 (m, 1H, DMTr), 6.93 (d, *J* = 8.8 Hz, 4H, DMTr), 5.52 (s, 1H, H-1′), 4.60 (s, 1H, H-2′), 4.37 – 4.32 (m, 2H, H-3′, C**H**$_2$–C≡CH), 4.29 (dd, *J* = 15.9, 2.4 Hz, 1H, C**H**$_2$–C≡CH), 3.75 (s, 6H, OCH_3), 3.72 – 3.70 (d, *J* = 8.0 Hz, 1H, H-5″), 3.69 – 3.68 (d, *J* = 8.0 Hz, 1H, H-5″), 3.58 (t, *J* = 2.4 Hz, 1H, C≡CH), 3.39 (d, *J* = 11.8 Hz, 1H, H-5′), 3.36 – 3.34 (m, 1H, H-5′, merged with H_2O signal from DMSO-*d*$_6$), 1.56 (d, *J* = 1.1 Hz, 3H, CH_3).^{13}C NMR (126 MHz, DMSO) δ 164.3 (C4), 158.7 (DMTr), 150.3 (C2), 145.0, 135.6, 135.4 (DMTr), 134.5 (C6), 130.25, 130.18, 128.5, 128.1, 127.3, 113.8 (DMTr), 109.1 (C5), 87.1 (C4′), 87.0 (C1′), 86.3 (DMTr), 80.2 (**C**≡CH), 78.6 (C≡**C**H), 76.5 (C2′), 75.8 (C3′), 72.1 (C5″), 58.4 (C5′), 57.4 (**C**H$_2$–C≡CH), 55.5 (OCH_3), 12.9 (CH_3).

Synthesis of 5′-*O*-(4,4′-dimethoxytrityl)-3′-O-propargyl-5-methyl LNA cytidine (7)

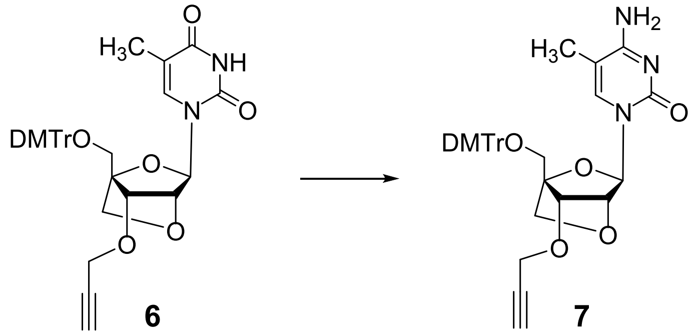

Nucleoside **6** (0.408 g, 0.668 mmol) was co-evaporated with anhydrous pyridine (3 × 10 mL) and re-dissolved in anhydrous pyridine (5 mL). The solution was cooled to 0 °C and N-methylimidazole (0.7 mL, 8.8 mmol) was added. The reaction mixture was stirred at 0 °C for 15 min, whereupon a freshly distilled $POCl_3$ (0.25 mL, 2.7 mmol) was added dropwise. The reaction was stirred at 0 °C for 30 min and then at room temperature for an additional 30 min. Concentrated aqueous ammonia (5 mL) was added and the reaction was stirred at room temperature for 16 h. The solvents were removed under reduced pressure. The residue was dissolved in CH_2Cl_2 (50 mL) and washed with brine (2 × 30 mL). The aqueous phase was back extracted with CH_2Cl_2 (2 × 30 mL). The combined organic phase was dried (Na_2SO_4), and concentrated under reduced pressure. The crude was then purified using column chromatography (0% to 7% MeOH/CH_2Cl_2) to obtain nucleoside **7** (0.233 g, 0.382 mmol, 57%) as a white foam. R_f = 0.5 (8% MeOH in CH_2Cl_2, v/v). ESI HRMS m/z 608.2406 ($[M-H]^-$, $C_{35}H_{34}O_7N_3^-$ calc. 608.2402. ^1H NMR (500 MHz, DMSO-d_6) δ 7.57 (s, 1H, H-6), 7.47 – 7.45 (m, 2H, DMTr), 7.41 (broad s, 1H, N-H), 7.37 – 7.31 (m, 6H, DMTr), 7.28 – 7.25 (m, 1H, DMTr), 6.93 (d, *J* = 8.8 Hz, 4H, DMTr), 6.85 (broad s, 1H, NH), 5.50 (s, 1H, H-1′), 4.56 (s, 1H, H-2′), 4.34 – 4.30 (m, 2H, H-3′, C**H**$_2$–C≡CH), 4.25 (dd, *J* = 16.0 Hz, 2.4 Hz, 1H, C**H**$_2$–C≡CH), 3.75 (s, 6H, OCH_3), 3.68 (s, 2H, H-5″), 3.56 (t, *J* = 2.4 Hz, 1H, C≡CH), 3.36 (s, 2H, H-5′, merged with H_2O signal from DMSO-d_6), 1.62 (s, 3H, CH_3).^{13}C NMR (126 MHz, DMSO) δ 166.0 (C4), 158.7 (DMTr), 155.1 (C2), 144.9 (DMTr), 136.8 (C6), 135.7, 135.5, 130.25, 130.18, 128.5, 128.2, 127.3, 113.83, 113.81 (DMTr), 101.4 (C5), 87.5 (C1′), 86.8 (C4′), 86.3 (DMTr), 80.1 (**C**≡CH), 78.6 (C≡**CH**), 76.5 (C2′), 75.5 (C3′), 72.0 (C5″), 58.5 (C5′), 57.4 (**CH$_2$**-C≡CH), 55.5 (OCH_3), 14.0 (CH_3).

Preparation of solid support carrying 5′-*O*-(4,4′-dimethoxytrityl)-3′-O-propargyl-5-methyl LNA cytidine (8)

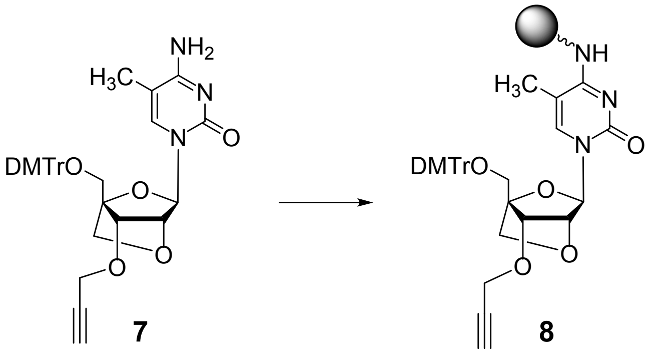

Amino-SynBase resin 500/100 (Link Technologies, Glasgow, UK) (500 Å pore size, loading 69 μmol/g, 4.06 g, 0.28 mmol of amine) was activated using 3% solution of trichloroacetic acid in CH_2Cl_2 for 3 h

in a stoppered glass vessel fitted with a sinter and tap. The solvents were removed by filtration and the support was washed with triethylamine:diisopropylethylamine (9:1), CH_2Cl_2, and diethyl ether. The support was dried under vacuum for 1 h and re-suspended in anhydrous pyridine (10 mL). A solution of succinic anhydride (0.813 g, 8.13 mmol) and DMAP (160 mg, 1.3 mmol) in anhydrous pyridine (5 mL) was added and the vessel was rotated at room temperature for 20 h. The solvents were removed by filtration, and the support was washed with pyridine, CH_2Cl_2, and diethyl ether and dried under high vacuum for 1 h. 500 mg of the activated resin was taken forward and soaked in 1 mL of anhydrous pyridine for 10 min. Diisopropyl carbodiimide (DIC) (93 µL, 0.60 mmol), 1-hydroxybenzotriazole (HOBT) (93 µL, 0.69 mmol), and compound **7** (86 mg, 0.14 mmol) were added to the reaction vessel, and the vessel was rotated for 20 h at room temperature. Pentachlorophenol (45 mg, 0.17 mmol) was added, and the vessel was rotated for an additional 3 h. The solvents were removed by filtration, and the support was washed with pyridine, CH_2Cl_2, and diethyl ether. Piperdine (10% in DMF, 2 mL) was added and the vessel was rotated for 5 min at room temperature. The solvent was removed by filtration and the support was washed with CH_2Cl_2 and diethyl ether. Capping reagent (oligonucleotide synthesis grade, acetic anhydride/pyridine/THF:N-methylimidazole in THF, 1:1, 2 mL) was added and the vessel was rotated at room temperature for 1 h. The solvent was removed by filtration, and the resin was washed with pyridine, CH_2Cl_2, and diethyl ether and dried under high vacuum overnight. Loading of nucleoside **7** on the support determined by cleaving the DMT group and was found to be 26 µmol/g.

Synthesis and Purification of oligonucleotides

Synthesis of DNA oligonucleotides

Standard DNA phosphoramidites, solid supports and reagents were purchased from Link Technologies and Applied Biosystems. LNA phosphoramidites were obtained from Exiqon. Automated solid phase synthesis of oligonucleotides (trityl off) was performed on an Applied Biosystems 394 synthesiser. Synthesis was performed on 1.0 µmole scale involving cycles of acid-catalyzed detritylation, coupling, capping, and iodine oxidation. Standard DNA phosphoramidites were coupled for 60 s while extended coupling time of 10 min was used for LNA phosphoramidites. Coupling efficiencies and overall synthesis yields were determined by the inbuilt automated trityl cation conductivity monitoring facility and were ≥98.0% in all cases. The oligonucleotides were then cleaved from the solid support and protecting groups from the nucleobase and backbone were removed by exposure to concentrated aqueous ammonium hydroxide for 60 min at room temperature followed by heating in a sealed tube for 5 h at 55 °C.

Synthesis of RNA oligonucleotides

2'-TBS protected RNA phosphoramidite monomers with *t*-butylphenoxyacetyl protection of the A, G and C nucleobases were used to assemble RNA oligonucleotides. Benzylthiotetrazole (BTT) was used as the coupling agent, *t*-butylphenoxyacetic anhydride as the capping agent and 0.1 M iodine as the oxidizing agent (Sigma-Aldrich). Coupling time of 10 min was used and coupling efficiencies of >97% were obtained. Cleavage of oligonucleotides from the solid support and protecting groups from the nucleobase and backbone were removed by exposure to concentrated aqueous ammonia/ethanol (3/1 v/v) for 2 h at room temperature followed by heating in a sealed tube for 2 h at 55˚C.

Removal of 2′-TBS protection of RNA oligonucleotides

After cleavage from the solid support and removal of the protecting groups from the nucleobases and phosphodiesters in ammonia/ethanol as described above, oligonucleotides were concentrated to a small volume *in vacuo*, transferred to 15 mL plastic tubes and freeze dried (lyophilised). The residue was dissolved in DMSO (300 µL) and triethylamine trihydrofluoride (300 µL) was added after which the reaction mixtures were kept at 65 °C for 2.5 h. Sodium acetate (3 M, 50 µL) and butanol (3 mL) were added with vortexing and the samples were kept at −80 °C for 30 min then centrifuged at 13,000 rpm at 4 °C for 10 min. The supernatant was decanted and the precipitate was washed twice with ethanol (0.75 mL) then dried under vacuum.

Purification of oligonucleotides (DNA or RNA)

The fully deprotected oligonucleotides were then purified by reverse-phase high performance liquid chromatography (HPLC) on a Gilson system using a Luna 10 µm C8(2) 100 Å pore Phenomenex column (250 × 10 mm) with a gradient of acetonitrile in triethylammonium bicarbonate (TEAB) over 20 min at a flow rate of 4 mL per minute. Buffer A: 0.1 M TEAB, pH 7.5; buffer B: 0.1 M TEAB, pH 7.5, with 50% acetonitrile were used. Elution was monitored by UV absorption between 260-295 nm.

Synthesis of 3'-alkyne-5-methyl dC oligonucleotides and 3'-alkyne-5-methyl LNA-C oligonucleotides

3'-Alkyne-5-methyl dC oligonucleotides were synthesized on 1.0 µmole scale using 5'-*O*-(4,4'-dimethoxytrityl)-3'-*O*-propargyl-5-methyldeoxycytidine solid support (33 µmole/g loading on AM polystyrene, Applied Biosystems).[2] The resin was packed into a twist column and the desired oligonucleotides were assembled and purified by standard phosphoramidite oligonucleotide synthesis (described above). 3'-Alkyne-5-methyl LNA-C oligonucleotides were synthesized by a similar procedure using the solid support **8**. Purified oligonucleotides were characterised by electrospray mass spectrometry. Mass spectra of oligonucleotides were recorded either using a Bruker micrOTOFTM II focus ESI-TOF MS instrument in ES^- mode or a XEVO G2-QTOF MS instrument in ES^- mode (Table S1).

Synthesis of 5′-azide modified oligonucleotides

Trityl off oligonucleotides were assembled at 1.0 µmole scale and were treated with a 0.5 M solution of methyltriphenoxyphosphonium iodide in DMF (1.0 mL) while attached to the solid support in a synthesis column.[3] The solution was periodically passed through the column using two 1 mL syringes for 20 min at room temperature. The resin was then washed several times with DMF. In a separate vessel 50 mg of sodium azide was taken up in 1 mL DMF and heated to 70 °C for 10 min. The mixture was allowed to cool to room temperature and the supernatant was passed back and forth through the synthesis column using two 1 mL syringes.[4] The synthesis column was left at 55 °C for 5 h and during this time the solution was occasionally passed back and forth. The column was then washed with DMF followed by acetonitrile and dried by the passage of a stream of argon. The resultant 5′-azide oligonucleotide was cleaved from solid support and deprotected by exposure to concentrated aqueous ammonium hydroxide for 60 min at room temperature followed by heating in a sealed tube for 5 h at 55 °C and purified as described above. Purified oligonucleotides were then characterised by mass spectrometry (Table S1).

Synthesis of 13-mer oligonucleotides incorporating a single triazole linkage

Representative Procedure: A mixture of 5′-azide oligonucleotide (130 nm) and 3′-alkyne oligonucleotide (100 nm) was freeze dried and re-dissolved in milli-Q water (250 µL). The solution was flushed with a stream of argon and to this was added an aqueous solution of $CuSO_4$ (20 µL, 100 mM), an aqueous solution of sodium ascorbate (40 µL, 500 mM), and tris-hydroxypropyltriazole ligand[5] (5 mg). The resulting mixture was degassed with a stream of argon and left at room temperature for 2 h with occasional shaking. Reagents were then removed by NAP-10 gel-filtration and the ligated triazole oligonucleotide was purified by HPLC (as described above) and characterized by mass spectrometry (Table S1).

Synthesis of 13-mer oligonucleotides incorporating two triazole linkages

Representative Procedure: A 5′-azide oligonucleotide, a 3′-alkyne oligonucleotide, a 5′-azide-3′-alkyne oligonucleotide and a splint (40 nm each) were mixed with NaCl (200 µL, 3 M). Milli-Q water was added to raise the total volume to 1940 µL. The mixture was annealed by heating to 80 °C and then cooling slowly to room temperature. The content was then kept at 4 °C for 1 h. $CuSO_4$ (aqueous, 20 µL, 100 mM), sodium ascorbate (aqueous, 40 µL, 500 mM), and tris-hydroxypropyltriazole ligand[5] (4 mg) were added. Thus a final concentration of 20 µM of each oligo in 300 mM NaCl and a final volume of 2 mL was obtained. The reaction mixture was left at 4 °C for 3 h and then at room temperature for 1 h. Reagents were then removed by NAP-10 gel-filtration and the ligated triazole oligonucleotide was purified by denaturing 20% polyacrylamide gel electrophoresis and characterized by mass spectrometry (Table S1). Splint used: 5′-dTTTTTT GCTAGAGAAGTCG TTTTTT (For ON8 and ON9), 5′-dTTTTTTGCTGGAGAGGTCGTTTTTT (for ON13 and ON14).

Synthesis of an 81-mer template incorporating a single LNA-triazole linkage

ON32 and ON18 (Table S1, 70 nm of each) and a splint (70 nm) were mixed with NaCl (200 µL, 3 M) and total volume was brought to 1940 µL by the addition of milli-Q water. The mixture was annealed by heating to 80 °C and then cooling slowly to room temperature. $CuSO_4$ (aqueous, 20 µL, 100 mM), sodium ascorbate (aqueous, 40 µL, 500 mM), and tris-hydroxypropyltriazole ligand[5] (4 mg) were added. The reaction mixture was left at room temperature for 3 h. Reagents were then removed by NAP-10 gel-filtration and the ligated triazole oligonucleotide was purified by denaturing 12% polyacrylamide gel electrophoresis, and characterized by mass spectrometry (ON15, table S1). Splint used: 5′-dTGTGTGCTAGCGATCTTA.

Table S1. Mass spec analysis of modified oligonucleotides

ON code	Sequence	Calc mass	Found mass
ON2	5′-dCGACG ^{Me}C**t**T^{L}TGCAGC	3978	3978
ON3	5′-dCGACG ^{Me}C**t**TTGCAGC	3950	3950
ON5	5′-dCGACG ^{Me}C^{L}**t**TTGCAGC	3978	3978
ON6	5′-dCGACG ^{Me}C^{L}**t**T^{L}TGCAGC	4006	4006
ON8	5′-dCGA^{Me}C**t**T^{L}TCT^{Me}C**t**T^{L}AGC	3971	3972
ON9	5′-dCGA^{Me}C**t**TTCT^{Me}C**t**TAGC	3915	3915
ON11	5′-dCGACG ^{Me}C**t**^{Me}C^{L}TGCAGC	3977	3977
ON12	5′-dCGACG ^{Me}C**t**^{Me}CTGCAGC	3949	3949
ON13	5′-dCGA^{Me}C**t**^{Me}C^{L}TCT^{Me}C**t**^{Me}C^{L}AGC	3969	3970

ON14	5′-dCGA^{Me}Ct^{Me}CTCT^{Me}Ct^{Me}CAGC	3913	3914
ON15	5′-dGCA TTC GAG CAA CGT AAG ATC G ^{Me}CtT^{L} AGC ACA CAA TCT CAC ACT CTG GAA TTC ACA CTG ACA ATA CTG CCG ACA CAC ATA ACC	24783	24781
ON16	5′-dCGACG^{Me}C-(alkyne)	1829	1829
ON17	5′-dCGA^{Me}C(3′-alkyne)	1210	1210
ON18	5′-dGCATTCGAGCAACGTAAGATCG ^{Me}C(3′-alkyne)	7110	7110
ON19	5′-dCGACG^{Me}C^{L}-(3′-LNA alkyne)	1857	1857
ON20	5′-dN$_3$-^{Me}C^{L}TGCAGC	2148	2148
ON21	5′-dN$_3$-T^{L}TGCAGC	2149	2149
ON22	5′-dN$_3$-^{Me}CTGCAGC	2120	2120
ON23	5′-dN$_3$-TTGCAGC	2121	2121
ON24	5′-dN$_3$-^{Me}CAGC	1197	1197
ON25	5′-dN$_3$-TAGC	1198	1198
ON26	5′-dN$_3$-^{Me}C^{L}AGC	1225	1225
ON27	5′-dN$_3$-T^{L}AGC	1226	1226
ON28	5′- dN$_3$-TTCT^{Me}C(3′-alkyne)	1506	1506
ON29	5′-dN$_3$-^{Me}CTCT^{Me}C(3′-alkyne)	1505	1505
ON30	5′-dN$_3$-T^{L}TCT^{Me}C(3′-alkyne)	1534	1534
ON31	5′-dN$_3$-^{Me}C^{L}TCT^{Me}C(3′-alkyne)	1533	1533
ON32	5′-dN$_3$-T^{L}AG CAC ACA ATC TCA CAC TCT GGA ATT CAC ACT GAC AAT ACT GCC GAC ACA CAT AAC C	17673	17673

t denotes triazole linkage

Ultraviolet melting studies

UV DNA melting curves were recorded in a Cary 4000 Scan UV-Visible Spectrophotometer using 3 µM of each oligonucleotide in a 10 mM phosphate buffer containing 200 mM NaCl at pH 7.0. Samples were annealed by heating to 85 °C (10 °C/min) and then slowly cooling to 20 °C (1 °C/min). As these six successive cycles (heating and cooling) were performed at a gradient of 1 °C/min, the change in UV absorbance at 260 nm was recorded. The melting temperature was calculated from the 1st derivative of the melting curve using in built software.

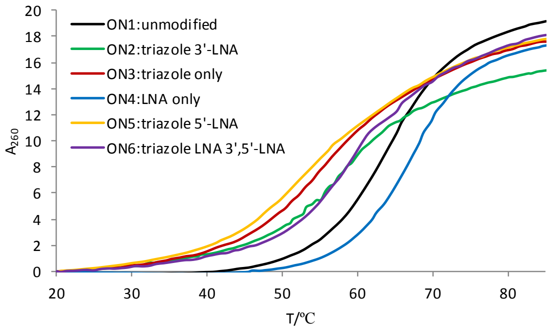

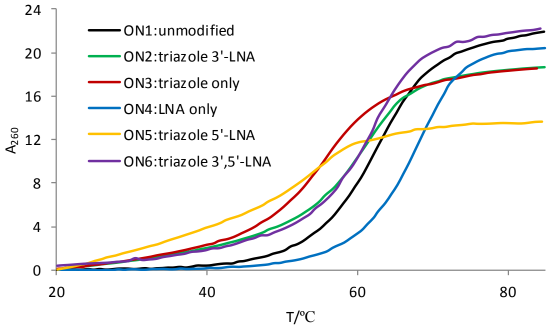

Figure S1: Representative melting curves for duplexes containing a single triazole linkage (^{Me}C-T step, left against DNA target and right against RNA target). For sequences see Table 1 (main manuscript).

Additional T_m data

Table S2. Thermal melting (T_m) data for duplexes incorporating a single triazole linkage (^{Me}C-^{Me}C step).

ON Code	ON Sequence	DNA target		RNA target	
		T_m[a]	ΔT_m[b]	T_m[a]	ΔT_m[b]
ON33	5′-d CGACG ^{Me}C**p**^{Me}CTGCAGC	68.7		69.1	
ON11	5′-d CGACG ^{Me}C**t**^{Me}C^{L}TGCAGC	63.5	−5.1	68.6	−0.5
ON12	5′-d CGACG ^{Me}C**t**^{Me}CTG CAGC	62.0	−6.4	63.4	−5.8
ON34	5′-d CGACG^{Me}C**p**^{Me}C^{L}TGCAGC	72.0	+3.3	74.7	+5.6

[a]Melting temperatures (T_m) were obtained from the maxima of the first derivatives of the melting curves (A_{260} vs. temperature) recorded in a buffer containing 10 mM phosphate and 200 mM NaCl at pH 7.0 using 3.0 µM concentrations of each strand. [b]ΔT_m = change in T_m for a modified duplex relative to the unmodified duplex (ON33), ^{Me}C is 5-methylcytosine, ^{Me}C^{L} is 5-methylcytosine LNA, **t** denotes a triazole linkage and **p** denotes a normal phosphodiester linkage. DNA target 5′-dGCT GCA GGC GTC G, RNA target 5′-rGCU GCA GGC GUC G.

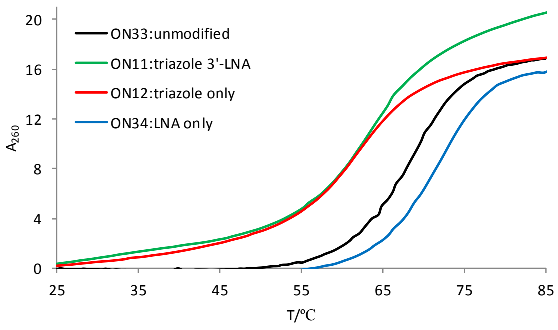

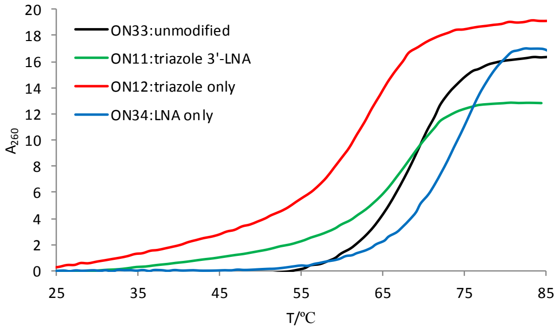

Figure S2: Representative melting curves for duplexes containing a single triazole linkage (^{Me}C-^{Me}C step, left against DNA target and right against RNA target). For sequences see Table S2.

Table S3. Mismatch discrimination of oligonucleotides incorporating a single triazole linkage (^{Me}C-T step) against RNA targets containing a mismatch nucleotide opposite the thymine nucleobase on 3′-side of the triazole linkage.

		RNA Target 3′-rGCUGCG<u>**M**</u>ACGUCG			
		T_m [a]	ΔT_m		
**ON Code**	**ON sequence**	**M = A**	**G**	**C**	**U**
ON1	5′-dCGACG ^{Me}C**p**TTGCAGC	62.8	−3.9	−16.3	−13.7
ON2	5′-dCGACG ^{Me}C**t**T^LTGCAGC	62.0	−3.3	−15.6	−13.4
ON3	5′-dCGACG ^{Me}C**t**TTG CAGC	56.6	−2.2	−16.1	−12.9
ON4	5′-dCGACG^{Me}C**p**T^LTGCAGC	68.9	−4.8	−15.2	−13.7

a) See table S2. ΔT_m = change in T_m relative to the fully matched duplex (**M** = A). ^{Me}C is 5-methylcytosine, $^{Me}C^L$ is 5-methylcytosine LNA, **t** denotes a triazole linkage and **p** denotes a normal phosphodiester linkage.

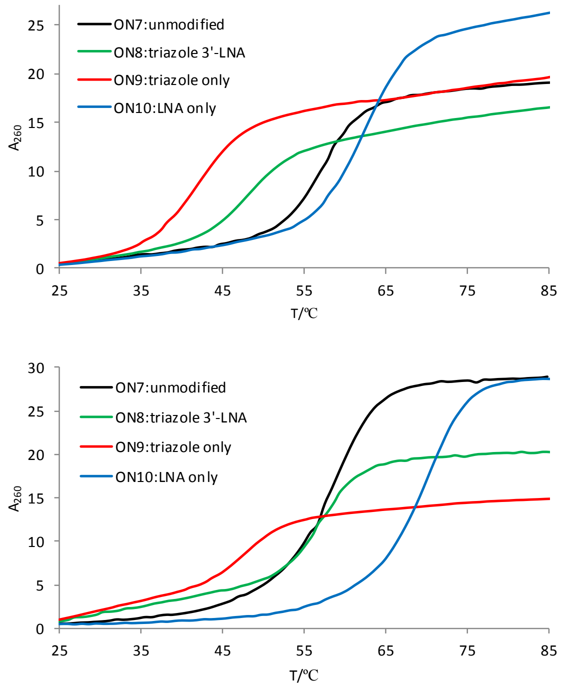

Figure S3: Representative melting curves for duplexes incorporating two triazole linkages (^{Me}C-T steps, left against DNA target and right against RNA target). For sequences see Table 2 (main manuscript).

Table S4. Thermal melting (T_m) data for duplexes incorporating two triazole linkages (^{Me}C-^{Me}C steps).

ON Code	Sequence	DNA target		RNA target	
		T_m[a]	ΔT_m/mod[b]	T_m[a]	ΔT_m/mod[b]
ON35	5′-dCGA^{Me}C**p**^{Me}CTCT^{Me}C**p**^{Me}CAGC	66.6		70.1	
ON13	5′-dCGA^{Me}C**t**^{Me}C^{L}TCT^{Me}C**t**^{Me}C^{L}AGC	56.4	−5.1	67.1	−1.5
ON14	5′-dCGA^{Me}C**t**^{Me}CTCT^{Me}C**t**^{Me}CAGC	51.9	−7.3	59.1	−5.5
ON36	5′-dCGA^{Me}C**p**^{Me}C^{L}TCT^{Me}C**p**^{Me}C^{L}AGC	72.2	+2.8	>75	>+2.5

a, b) see Table S2 footnote. DNA target: 5′-dGCT GGA GAG GTC G, RNA target: 5′-rG CUA GAG AAG UC G

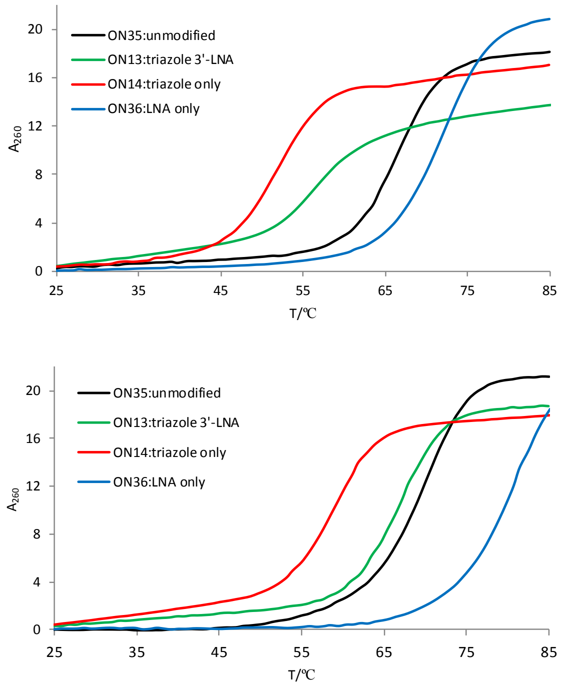

Figure S4: Representative melting curves for duplexes incorporating two triazole linkages (^{Me}C-^{Me}C steps, left against DNA target and right against RNA target). For sequences see Table S4.

CD spectroscopy

CD spectra (200−340 nm) were recorded on a Chirscan Plus spectropolarimeter using a Quartz optical cells with a path length of 3.0 mm. Scans were performed at 20 °C using a step size of 0.5 nm, a time per point of 1.0 s and a bandwidth of 2 nm, and the average of four scans is presented. Samples from UV melting studies (3 µM of each oligonucleotide in a 10 mM phosphate buffer containing 200 mM NaCl at pH 7.0) were used directly and were annealed by heating to 85 °C and then slowly cooled to 20 °C prior to recording CD spectrum. The average trace was smoothed (20 points) using in built software. A CD spectrum of only buffer was also recorded and was subtracted from the collected data. Finally, spectra were baseline-corrected using the offset at 340 nm.

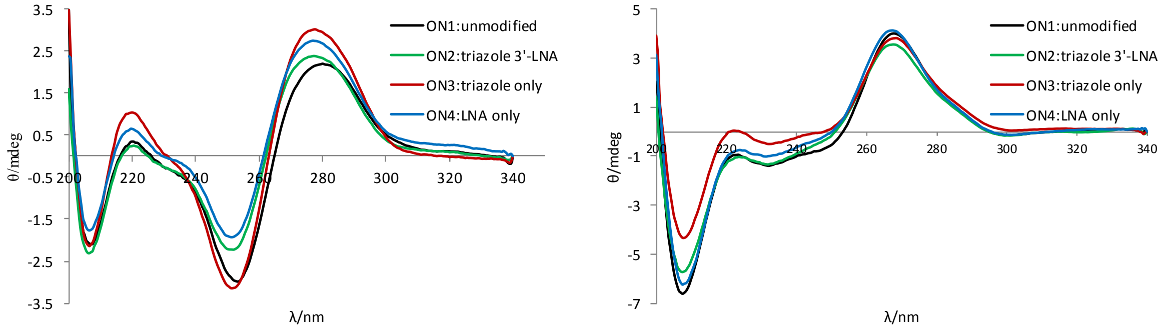

Figure S5: Representative CD curves for duplexes containing a single triazole linkage (^{Me}C-T step, left against DNA target; right against RNA target). For sequences see Table 1 (main manuscript).

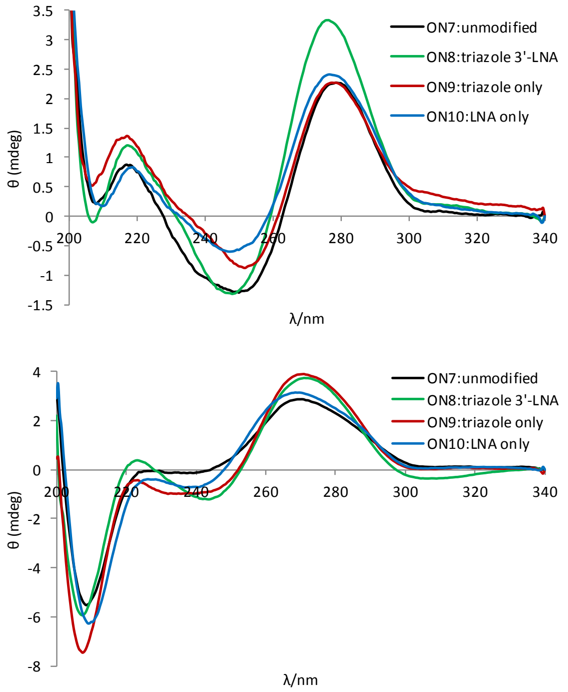

Figure S6: Representative CD curves for duplexes incorporating two triazole linkages (^{Me}C-T step, left against DNA target; right against RNA target). For sequences see Table 2 (main manuscript).

Snake venom phosphodiesterase stability

5 nm of oligonucleotide was dissolved in 50 µL buffer (100 mM Tris-HCl, 20 mM $MgCl_2$, pH = 9.0). 10 µL of this solution was removed as a control (zero min.) and was diluted with H_2O (10 µL). To the remaining solution was added 30 µL H_2O followed by 10 µL aqueous solution of Phosphodiesterase 1 from *Crotalus adamanteus* venom (from Sigma Aldrich, catalogue number P3243, 0.45 units, dissolved in 700 µL H_2O). The reaction was incubated at 37°C and aliquots (20 µL) were taken at different time intervals, mixed with formamide (20 µL) and stored at −20 °C. The samples were then analysed by denaturing 20% polyacrylamide gel electrophoresis.

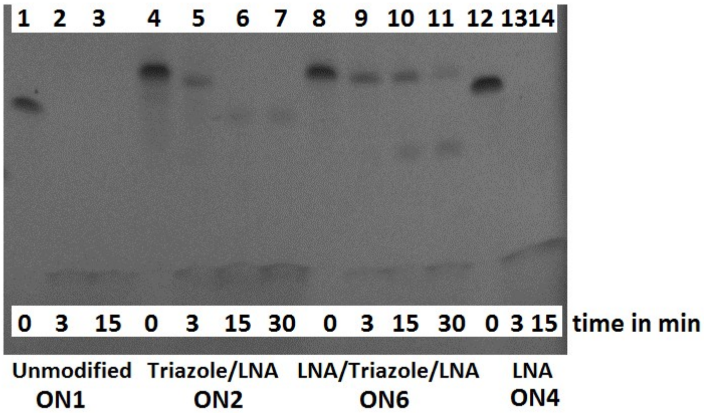

Figure S7. LNA triazole stabilises ON's to 3′-exonuclease digestion. The ON1:unmodified (lanes 1-3) and ON2:triazole 3′-LNA (lanes 4-7), ON6:triazole 3′,5′-LNA (lanes 8-11), ON4:LNA only (lane 12-14).

Linear copying of an 81-mer template incorporating a single LNA-triazole linkage

A reaction mixture was prepared by mixing 10 µL of 10 X NEB buffer 2* in a total reaction volume of 100 µL with template, primer or template + primer (110 pmol of each), 0.2 mM dNTP and 1.0 µL of DNA polymerase 1, Large Klenow fragment (5u/µL). Reaction mixture was left at 37°C for 2.5 h. Phenol:chloroform:isoamyl alcohol (25:24:1, v/v) solution (100 µL) was added and mixture was vortexed for 30 seconds, centrifuged for 5 min at 5000 rpm. Aqueous phase was collected and sodium acetate (10 µl, 3 M, pH 5.2) and ethanol (330 µL) were added. The mixture was left at −80 °C for 4 h and then centrifuged (13000 rpm) for 20 min at 4 °C. The supernatant was removed and the resulting pellet was dissolved in 20 µL H_2O. 10 µL sample was used for mass and another 10 µL was analysed by denaturing 10% polyacrylamide gel electrophoresis (Figure S8). Similar gels were obtained when reaction mixture was directly (prior to precipitation) loaded on the gel. Incubation of reaction mixture for 1.5 h showed truncated product in addition to full length product presumably stalling the reaction at the triazole step. The product was analysed by mass spectrometry.

**(10 X NEB buffer2 was supplied with the enzyme).1 X NEB buffer 2 = 50 mM NaCl, 10 mM Tris-HCl, 10 mM $MgCl_2$, 1 mM DTT (pH 7.9 at 25 °C).*

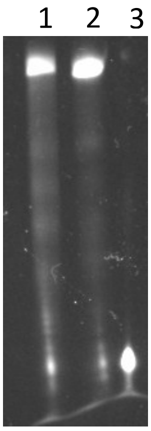

Figure S8: 10% denaturing polyacrylamide gel from linear copying reaction. Lane 1; Linear copying reaction using modified template (ON15) 5′-dGCA TTC GAG CAA CGT AAG ATC $G^{Me}C$tT^{L} AGC ACA CAA TCT CAC ACT CTG GAA TTC ACA CTG ACA ATA CTG CCG ACA CAC ATA ACC where t represent triazole linkage and T^{L} is LNA thymidine. Lane 2; Linear copying reaction using unmodified template 5′-dACGTTAGCACGAAGAGGCATCTTAGCACACAATCTCACACTCTGGAATTCACACTGACAATACTCGCGAACACACCCAAT. Lane 3; negative control: linear copying reaction using modified template without enzyme. For modified template: Full length product mass; found 26025, calc. 26025. A relatively small peak at 26337 (full length + A) was also observed. For unmodified template: Full length product mass; found 25695, calc. 25695. No M+A product was observed for control. Primer used: 5′-dFTGGTTATGTGTGTCGGCAG (for modified template), 5′-dFTATTGGGTGTGTTCGCGAG (for unmodified template), F is amidohexylfuorescein.

PCR of an 81-mer template incorporating a single LNA-triazole linkage

PCR amplification of the modified template (ON15) was achieved using GoTaq DNA polymerase. 10 µL of 5X buffer (Promega gree PCR buffer) was used in a total reaction volume of 50 µL with 12.5 ng of the DNA template, 0.5 µM of each primer, 0.2 mM dNTP and 1.25 unit of GoTaq polymerase. PCR cyclic conditions: 95 °C (initial denaturation) for 2 min then 3 cycles of 95 °C (denaturation) for 15 s, 54 °C (annealing) for 20 s and 72 °C extension for 5 min. Next 20 cycles 95 °C (denaturation) for 15 s, 54 °C (annealing) for 20 s and 72 °C extension for 30 s. This was followed by leaving the PCR reaction mixture at 72 °C for 5 min. The PCR amplicon was analysed by loading onto 2% agarose gel, and was precipitated following the procedure described for linear copying for mass analysis. Primers used: 5′ -dGCATTCGAGCAACGTAAG, 5′-dGGTTATGTGTGTCGGCAG (for modified

template). The unmodified template 5′-dACGTTAGCACGAAGAGGCATCTTAGCACACAATCTCACACTCTGGAATTCACACTGACAATACTCGCGAACACACCCAAT was used as a control. Primers used: 5′-dATTGGGTGTGTTCGCGAG, 5′-dACGTTAGCACGAAGAGGC. Mass analysis for control: [M+A] strand 1: Calc. 24764, found 24765. Strand 2: Calc.25167, found 25168.

UV trace from HPLC of HPLC/mass spec for modified oligonucleotides

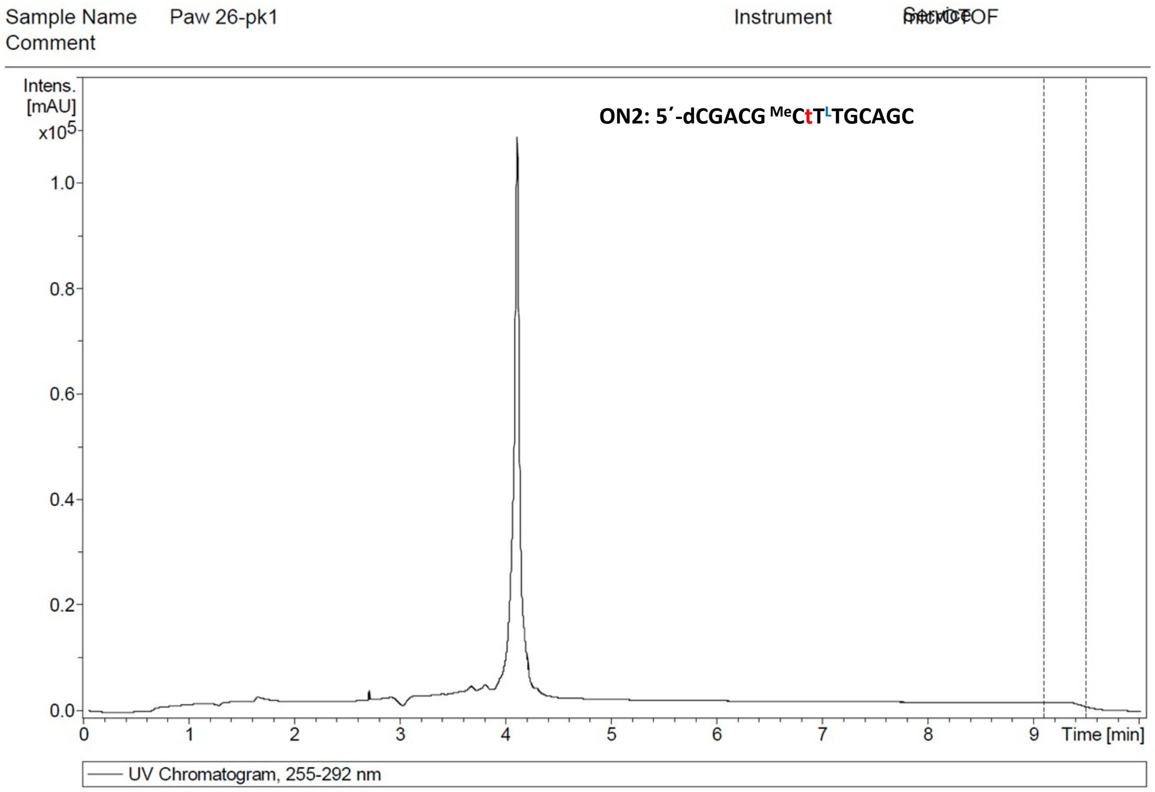

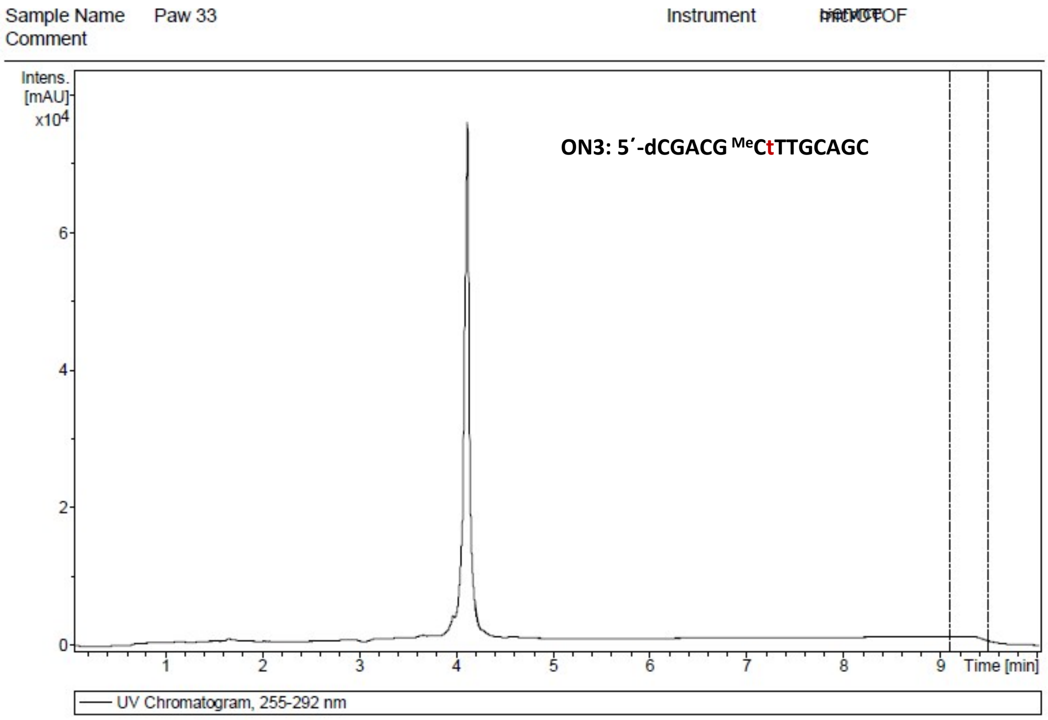
Sample Name
Paw 33
Instrument
Comment
Intens. [mAU] x10^4
ON3: 5´-dCGACG MeCtTTGCAGC
Time [min]
UV Chromatogram, 255-292 nm

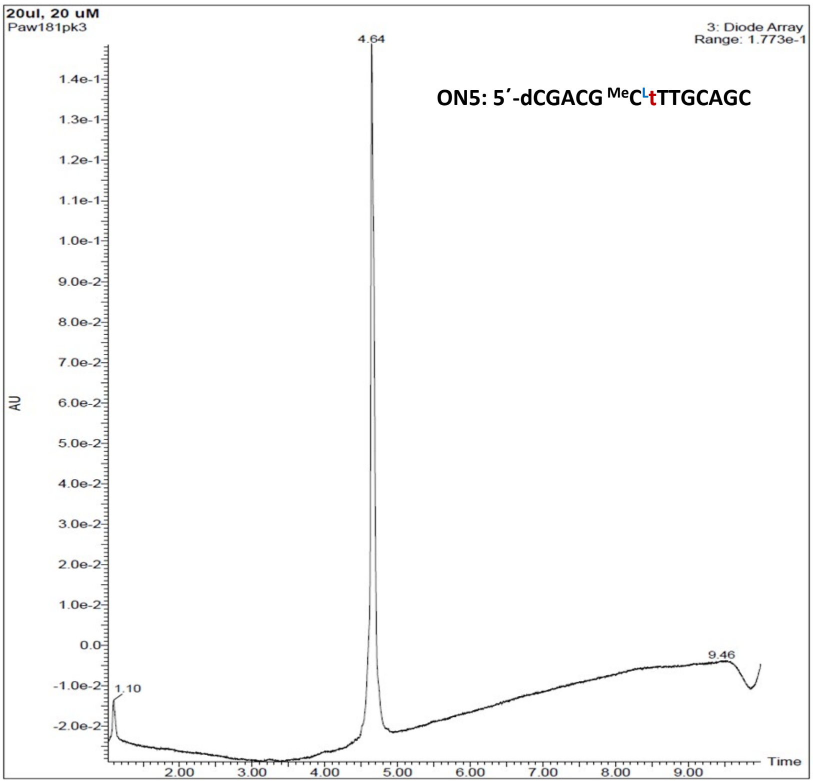
20ul, 20 uM
Paw181pk3
3: Diode Array
Range: 1.773e-1
4.64
ON5: 5´-dCGACG MeCLtTTGCAGC
AU
1.10
9.46
Time

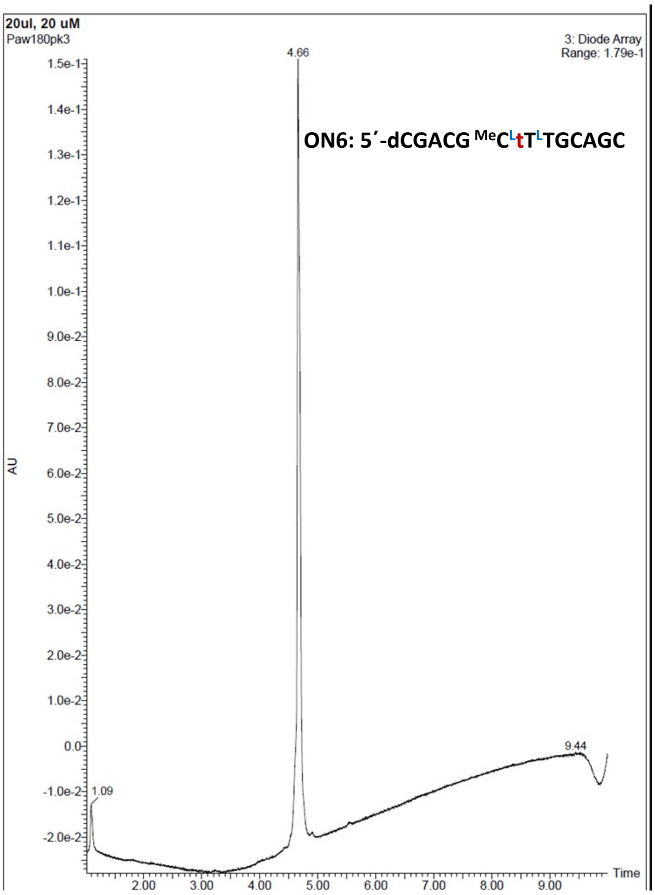
20ul, 20 uM
Paw180pk3
3: Diode Array
Range: 1.79e-1
ON6: 5′-dCGACG ^{Me}C^{L}tT^{L}TGCAGC
4.66
1.09
9.44
AU
Time

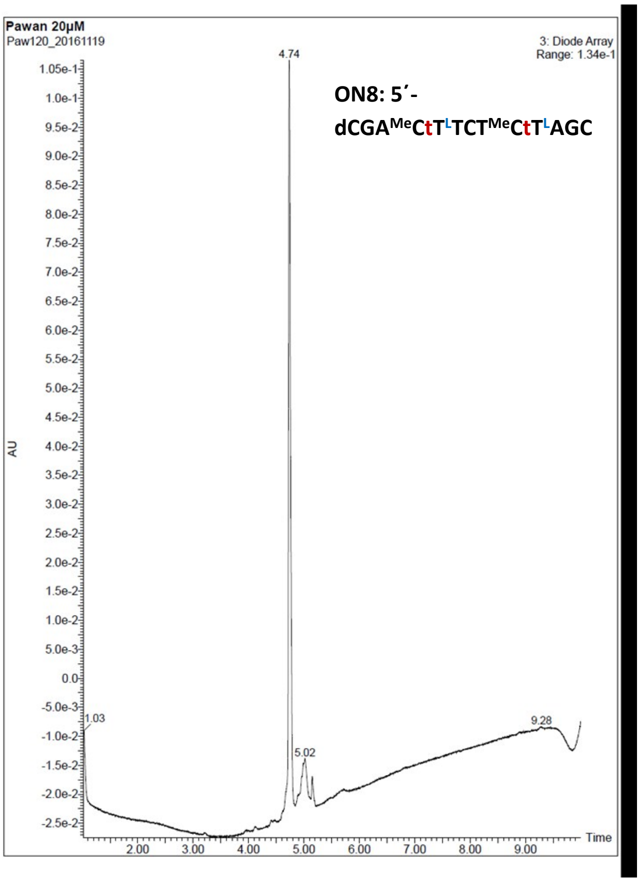
Pawan 20µM
Paw120_20161119
3: Diode Array
Range: 1.34e-1
ON8: 5′-
dCGA^{Me}CtT^{L}TCT^{Me}CtT^{L}AGC
4.74
5.02
1.03
9.28
AU
Time

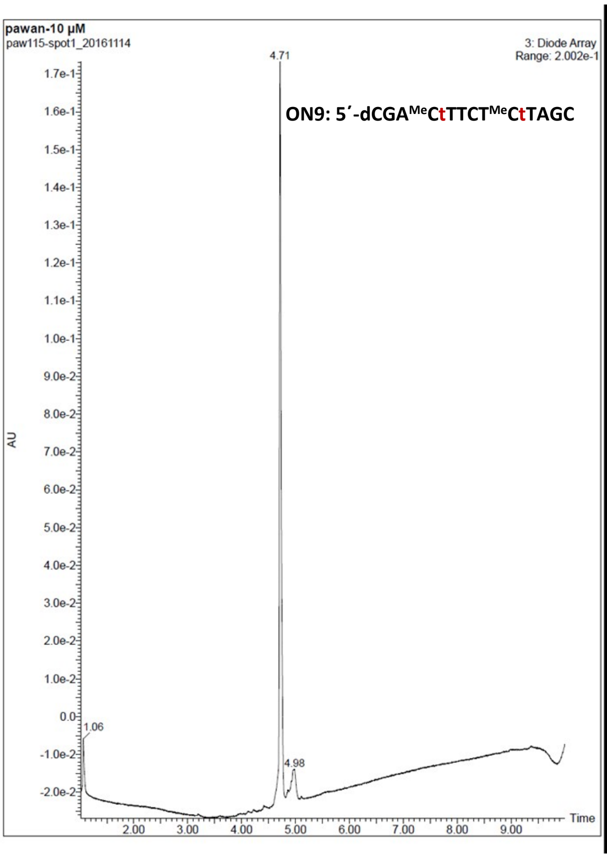
pawan-10 µM
paw115-spot1_20161114
3: Diode Array
Range: 2.002e-1
4.71
ON9: 5´-dCGA^{Me}CtTTCT^{Me}CtTAGC
1.06
4.98
AU
Time

Comment

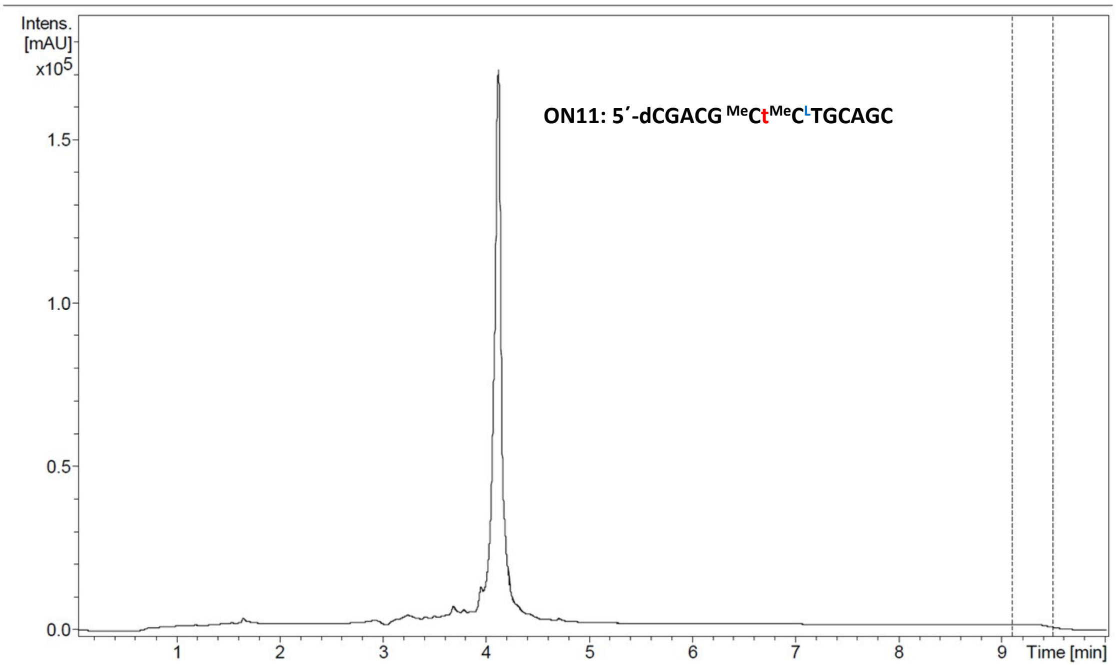
Intens.
[mAU]
x10^{5}
ON11: 5´-dCGACG ^{Me}Ct^{Me}C^{L}TGCAGC
Time [min]

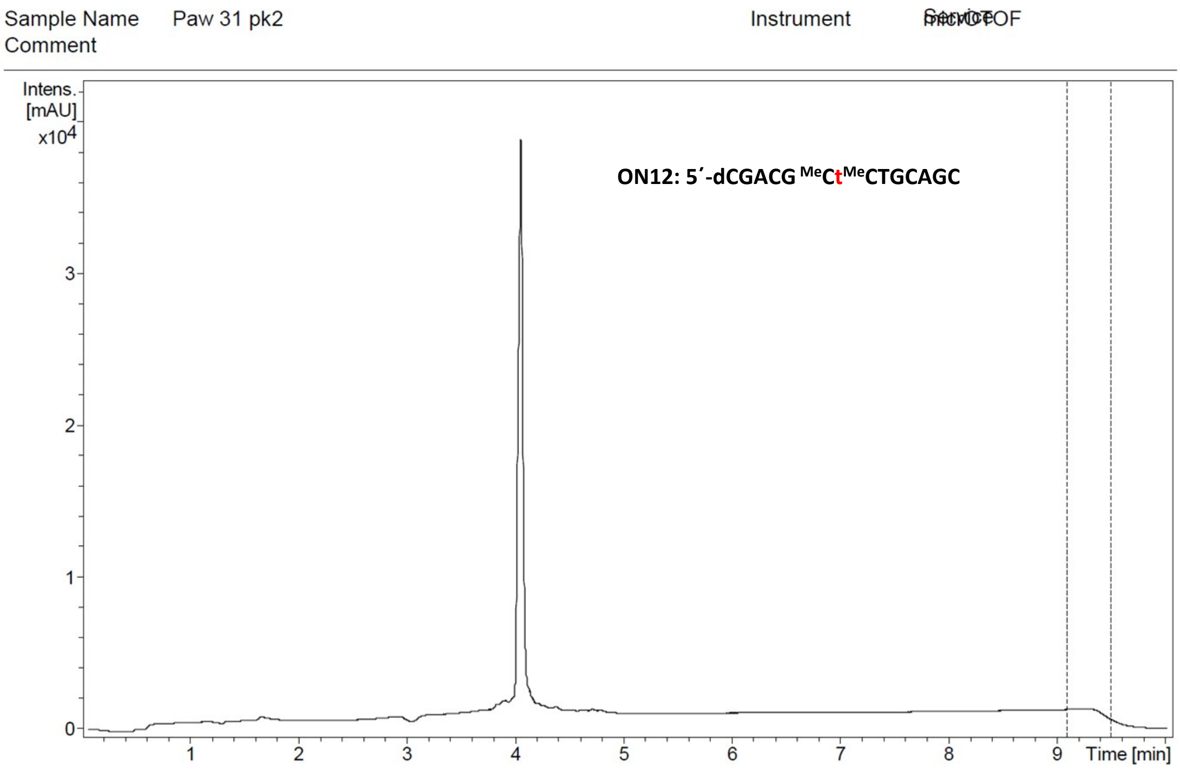
Sample Name Paw 31 pk2
Comment
Instrument
Intens. [mAU] x10^4
ON12: 5´-dCGACG ^{Me}C**t**^{Me}CTGCAGC
Time [min]

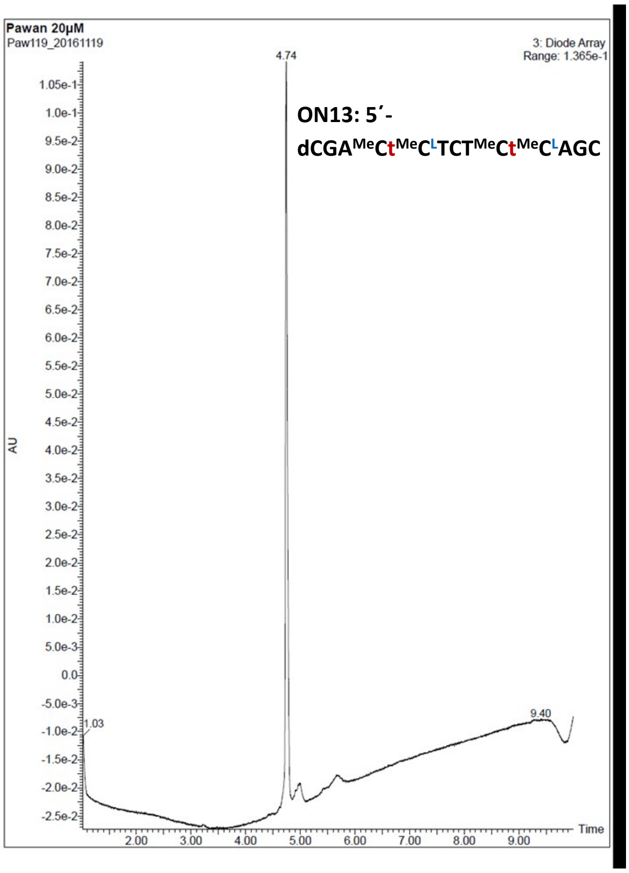
Pawan 20µM
Paw119_20161119
3: Diode Array
Range: 1.365e-1
4.74
ON13: 5´-dCGA^{Me}C**t**^{Me}C^{L}TCT^{Me}C**t**^{Me}C^{L}AGC
AU
1.03
9.40
Time

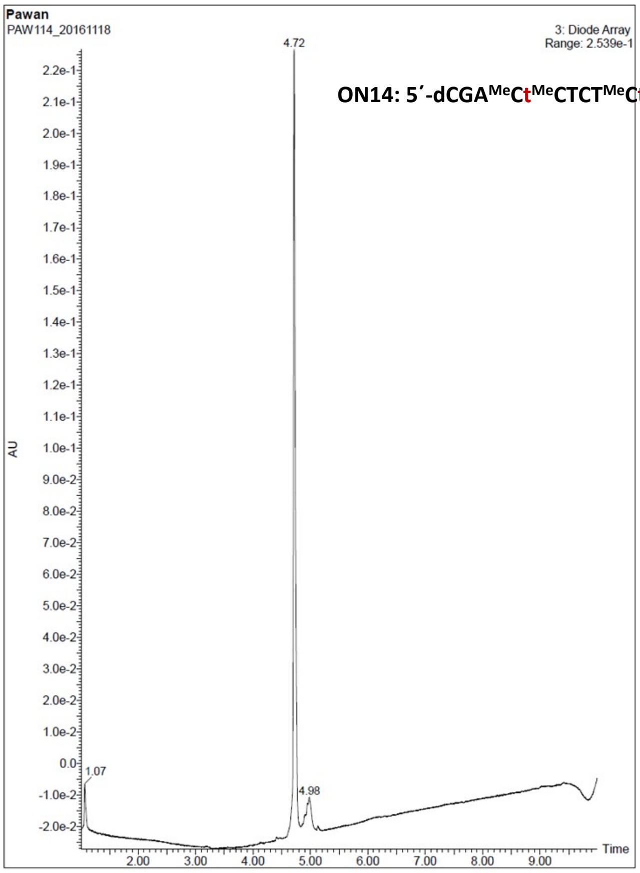

ON14: 5´-dCGA^{Me}Ct^{Me}CTCT^{Me}Ct^{Me}CAGC

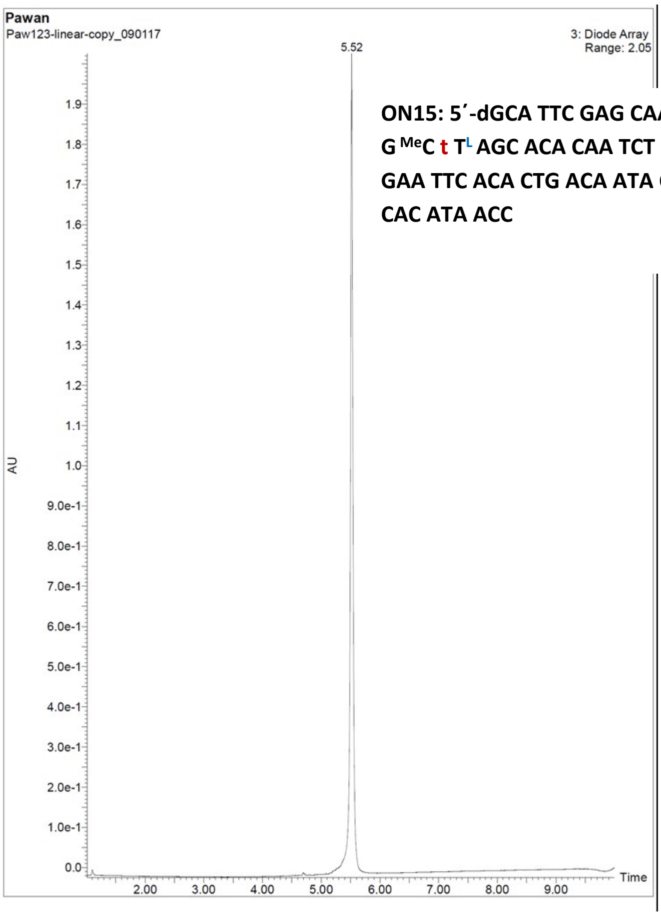

ON15: 5´-dGCA TTC GAG CAA CGT AAG ATC G ^{Me}C t T^{L} AGC ACA CAA TCT CAC ACT CTG GAA TTC ACA CTG ACA ATA CTG CCG ACA CAC ATA ACC

NMR Spectra

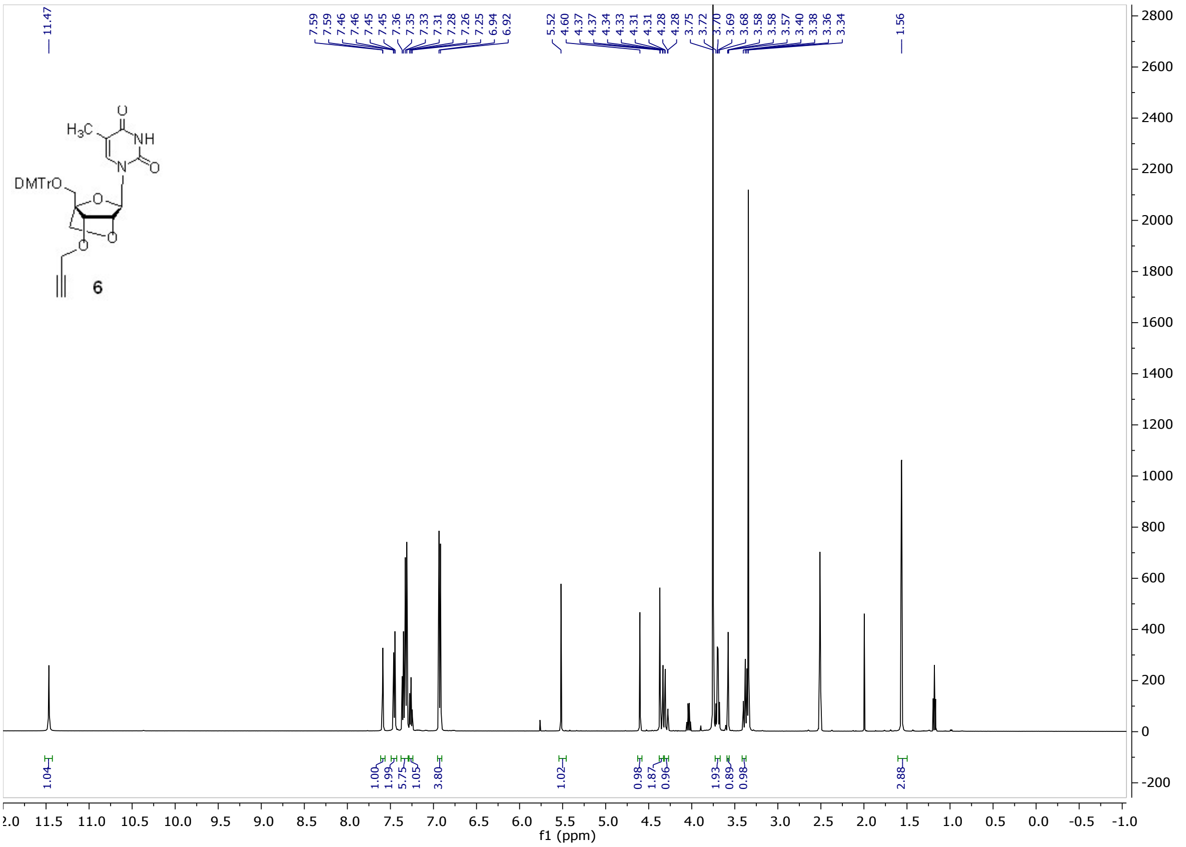

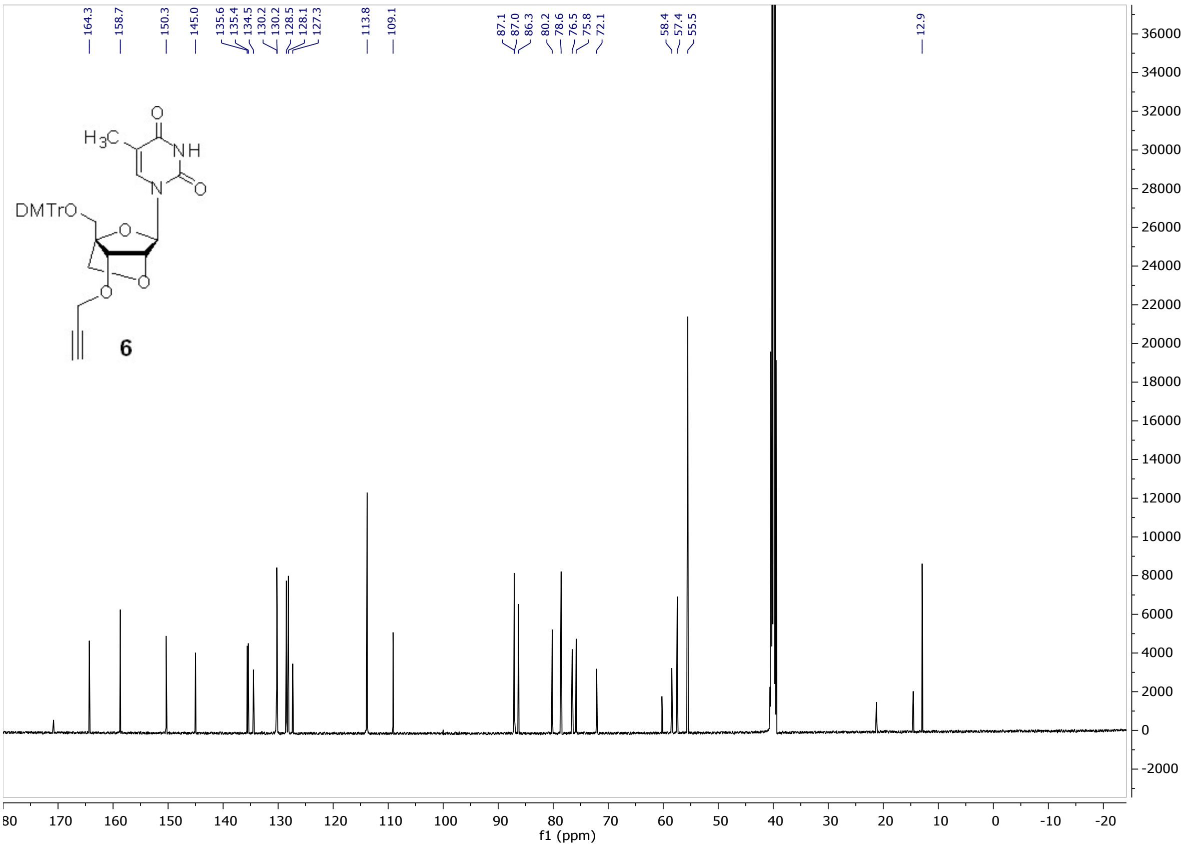

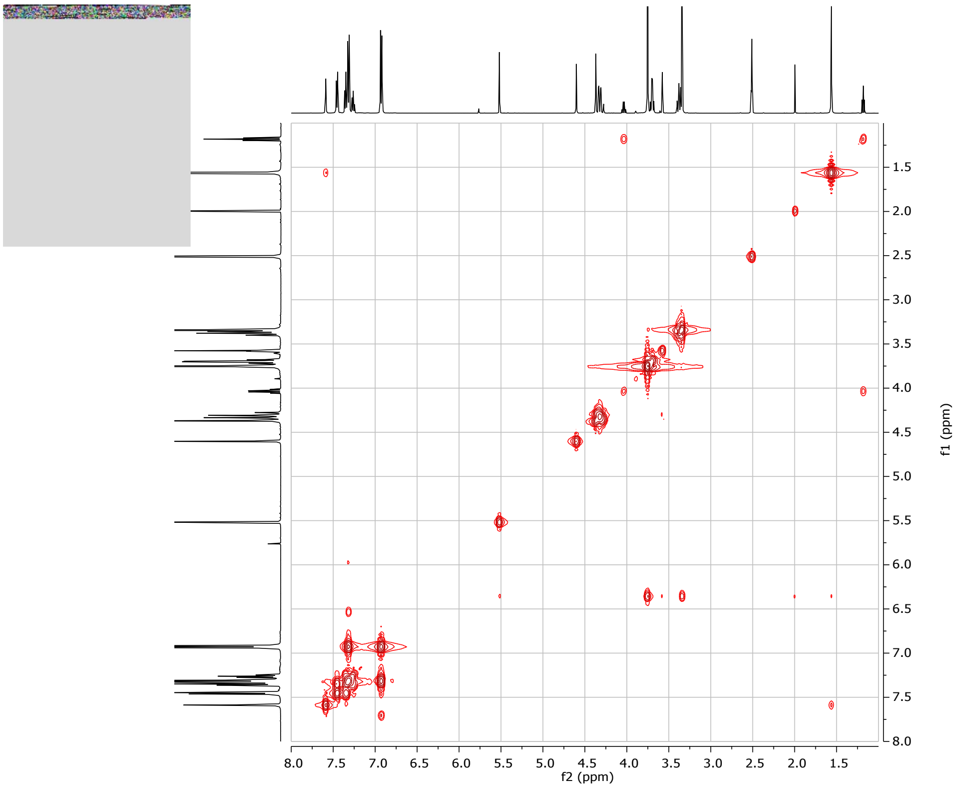
f2 (ppm)
f1 (ppm)

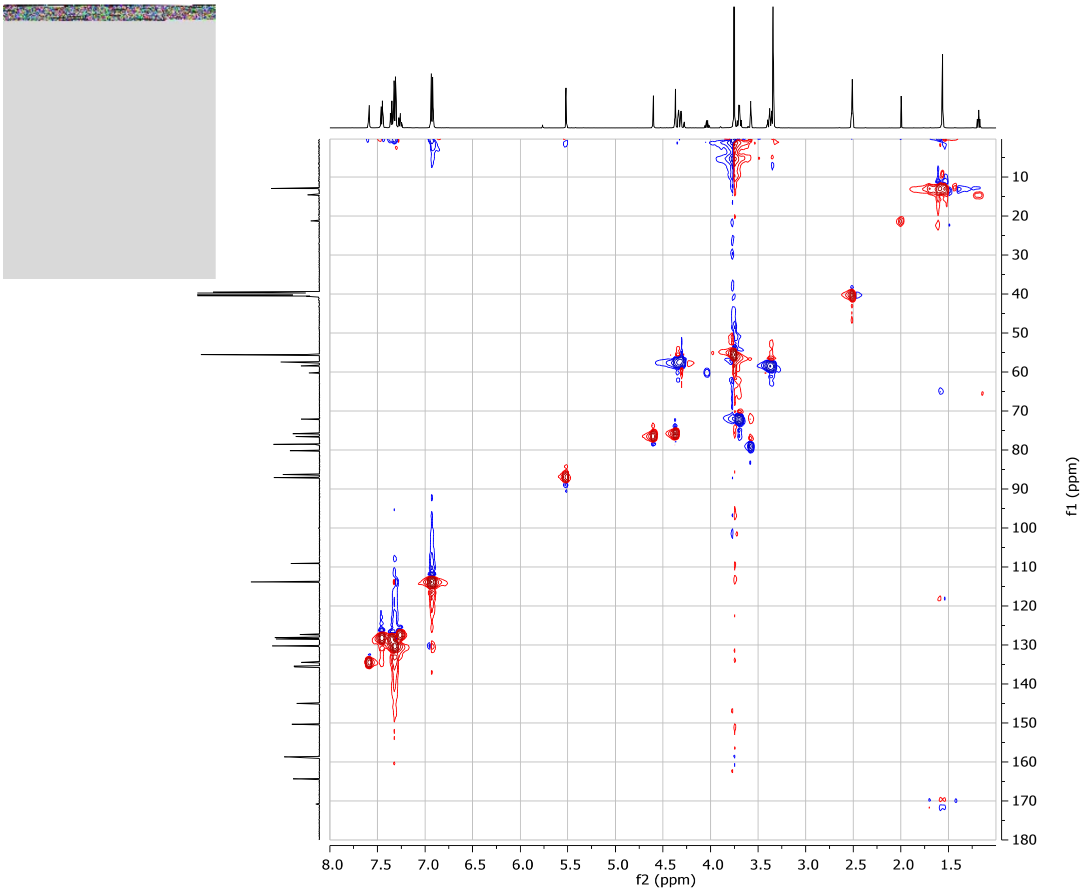
f2 (ppm)
f1 (ppm)

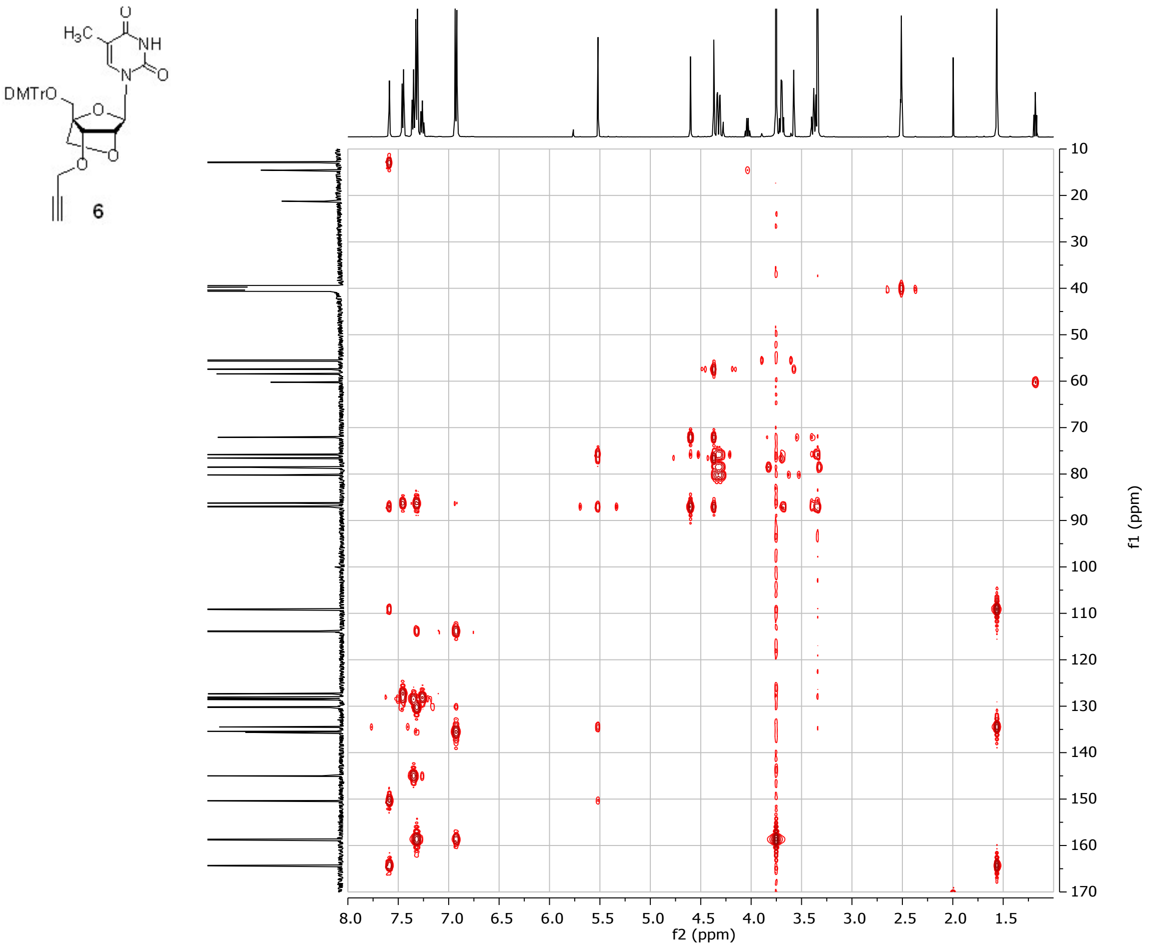
DMTrO
6
f2 (ppm)
f1 (ppm)

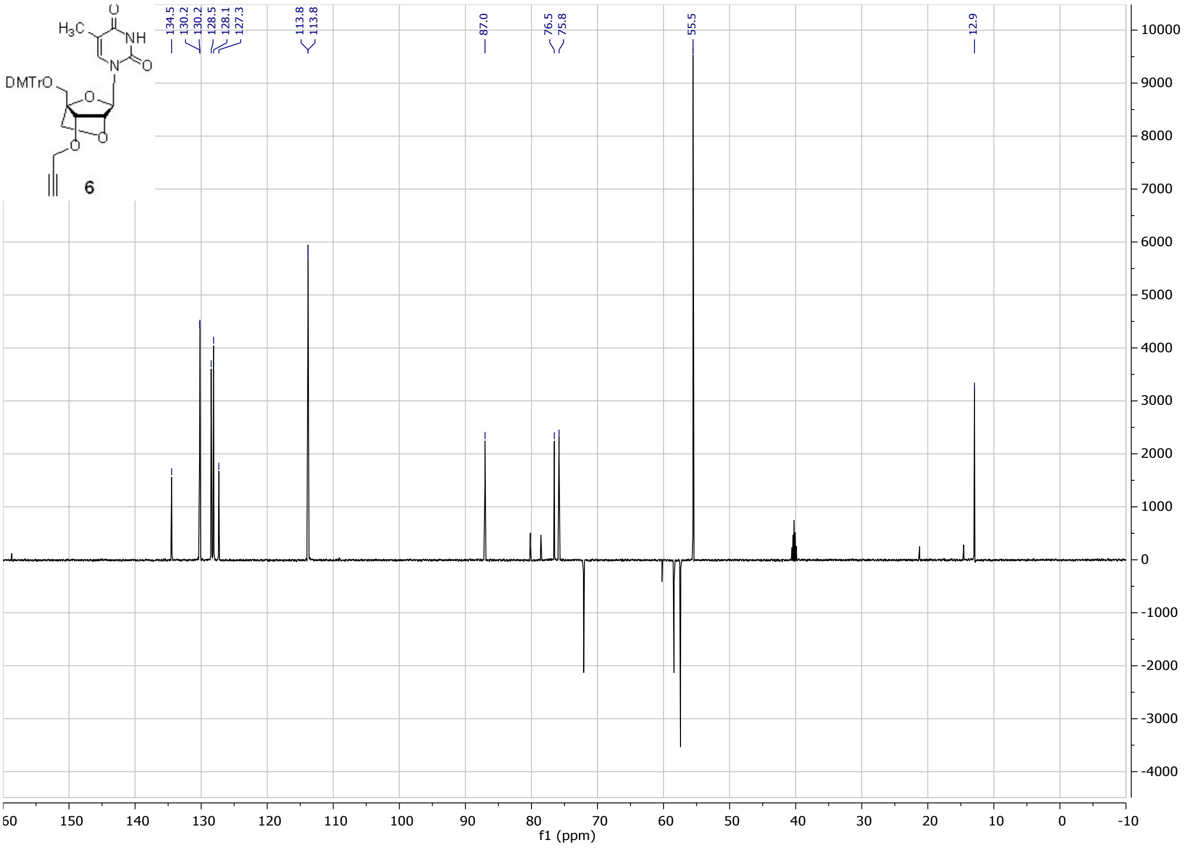
DMTrO
6
134.5
130.2
130.2
128.5
128.1
127.3
113.8
113.8
87.0
76.5
75.8
55.5
12.9
f1 (ppm)

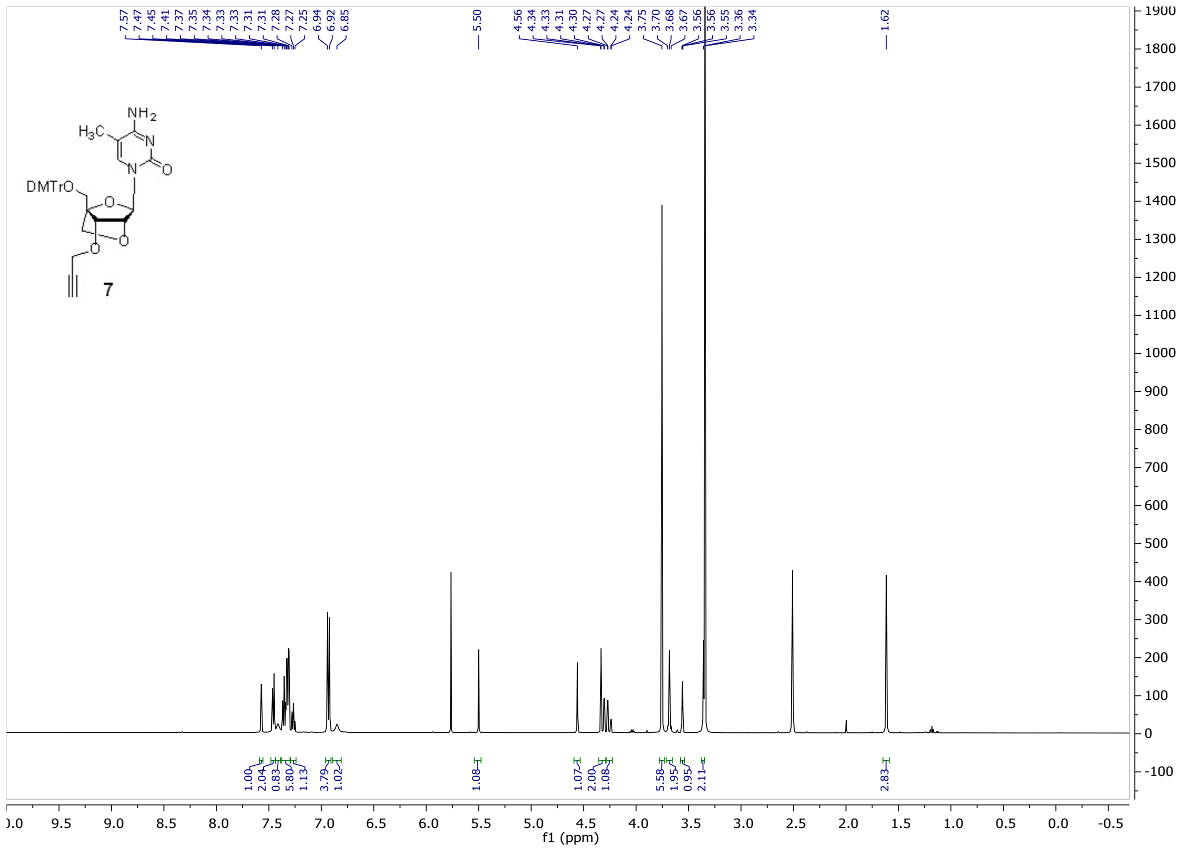

7.57 7.47 7.45 7.41 7.37 7.35 7.34 7.33 7.33 7.31 7.31 7.28 7.27 7.25 6.94 6.92 6.85
5.50
4.56 4.34 4.33 4.31 4.30 4.27 4.27 4.24 4.24 3.75 3.70 3.68 3.67 3.56 3.56 3.55 3.36 3.34
1.62
NH2
H3C
N
O
DMTrO
7
1.00 2.04 0.83 5.80 1.13 3.79 1.02 1.08 1.07 2.00 1.08 5.58 1.95 0.95 2.11 2.83
f1 (ppm)

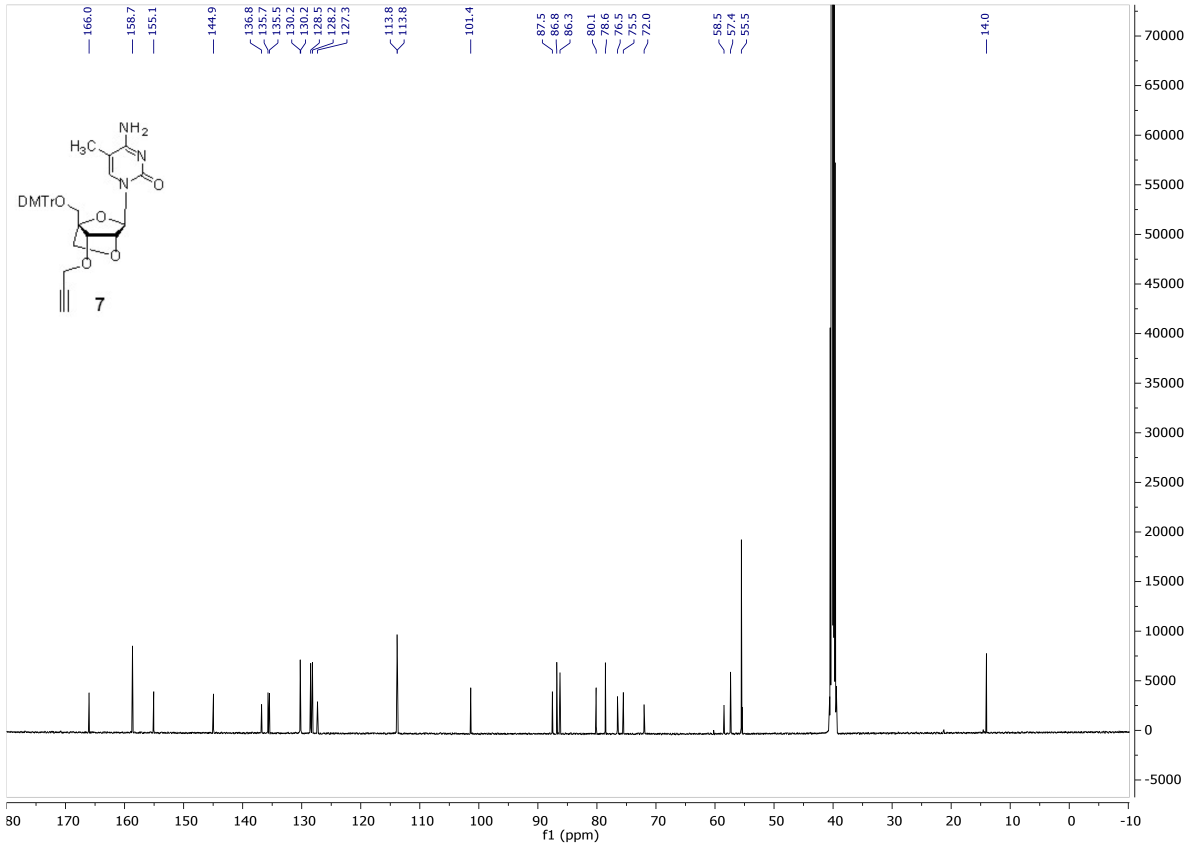

166.0 158.7 155.1 144.9 136.8 135.7 135.5 130.2 130.2 128.5 128.2 127.3 113.8 113.8 101.4 87.5 86.8 86.3 80.1 78.6 76.5 75.5 72.0 58.5 57.4 55.5 14.0
NH2
H3C
N
O
DMTrO
7
f1 (ppm)

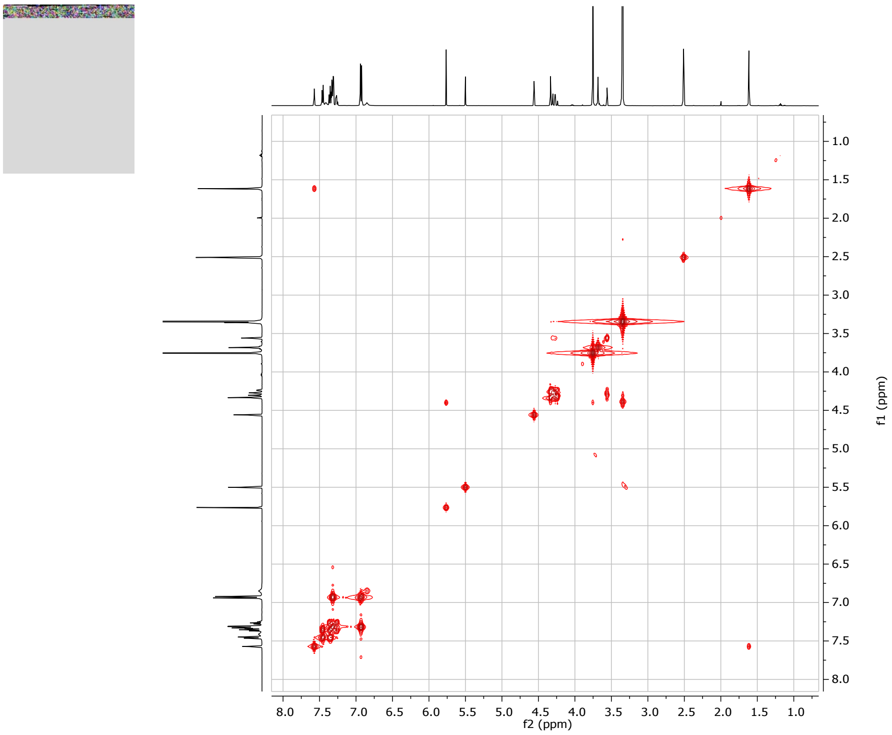
f2 (ppm)
f1 (ppm)

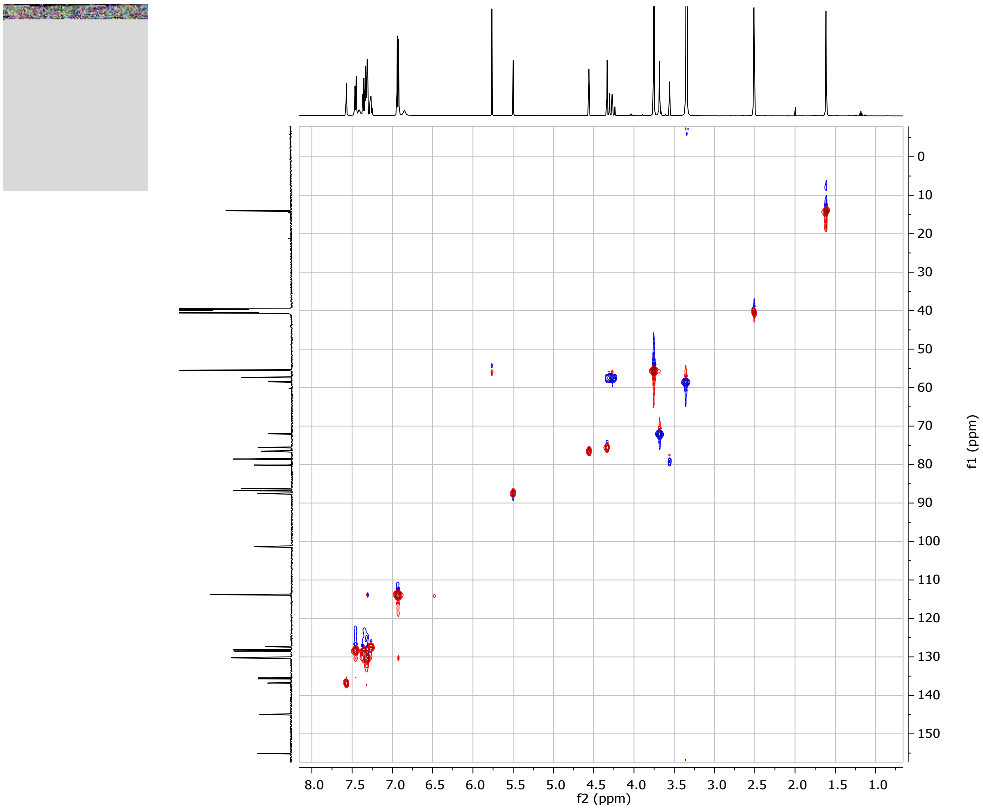
f2 (ppm)
f1 (ppm)

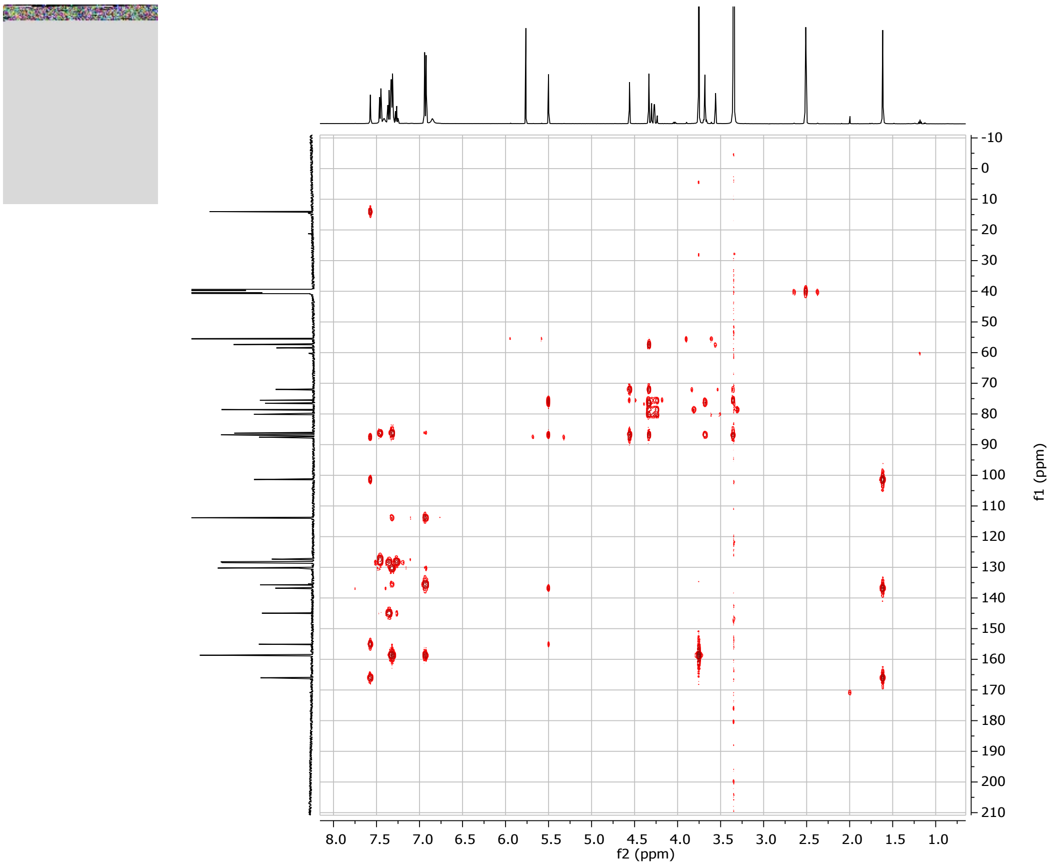

f2 (ppm)
f1 (ppm)

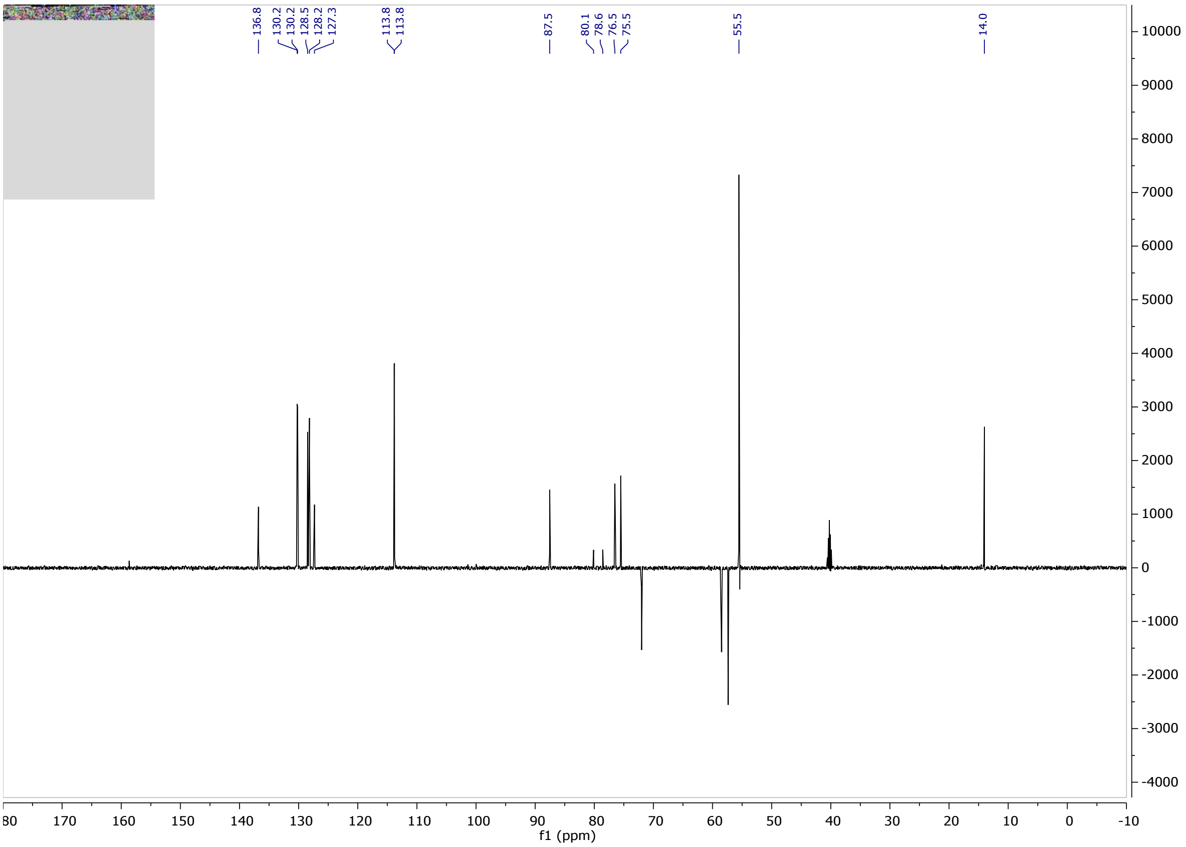

136.8
130.2
130.2
128.5
128.2
127.3
113.8
113.8
87.5
80.1
78.6
76.5
75.5
55.5
14.0
f1 (ppm)

References

1. S. Obika, T. Uneda, T. Sugimoto, D. Nambu, T. Minami, T. Doi, T. Imanishi, Bioorg Med Chem 2001, 9, 1001-1011.

2. A. H. El-Sagheer, A. P. Sanzone, R. Gao, A. Tavassoli and T. Brown, Proc Natl Acad Sci U S A, 2011, 108, 11338-11343.

3. G. P. Miller and E. T. Kool, Org Lett, 2002, 4, 3599-3601.

4. G. P. Miller and E. T. Kool, J Org Chem, 2004, 69, 2404-2410.

5. T. R. Chan, R. Hilgraf, K. B. Sharpless and V. V. Fokin, Org Lett, 2004, 6, 2853-2855.
